# Laser-Engineered Ti/RuO_2_–Sb_2_O_4_–TiO_2_ Anodes Enabling Energy-Efficient Electrochemical Degradation of Thiamethoxam in Flow Reactors

**DOI:** 10.1021/acsomega.6c07684

**Published:** 2026-09-28

**Authors:** Aline R. Dória, Géssica O. S. Santos, Giancarlo R. Salazar-Banda, Katlin I. B. Eguiluz, Luís A. M. Ruotolo

**Affiliations:** † Department of Chemical Engineering, Federal University of São Carlos, Rod. Washington Luiz, km 235, São Carlos, São Paulo 13565-905, Brazil; ‡ Graduate Program in Process Engineering (PEP), Tiradentes University, Aracaju, Sergipe 49032-490, Brazil; § Electrochemistry and Nanotechnology Laboratory, Institute of Technology and Research (ITP), Aracaju, Sergipe 49032-490, Brazil

## Abstract

In this study, Ti/RuO_2_–Sb_2_O_4_–TiO_2_ anodes were synthesized using innovative rapid thermal treatment methods based on CO_2_ laser irradiation and hybrid microwave heating. Structural characterizations demonstrated that these rapid heating methods led to coatings that were more homogeneous, with reduced exposure of the Ti substrate, compared to conventional furnace calcination. The electrochemical responses showed that anode preparation using the laser and microwave methods resulted in an approximately 2-fold higher electroactive area and a 3-fold lower charge-transfer resistance, compared to the electrode prepared by the furnace method. Furthermore, the electrodes produced by the nonconventional methods showed markedly improved service lifetimes, highlighting the laser-prepared anode (37 h), with an increase of around 1 order of magnitude, compared to the furnace-prepared electrode. Based on this superior performance, the laser-prepared anode was scaled up from 2 to 56 cm^2^ and was applied in the electrooxidation of thiamethoxam (TMX) in a recirculating filter-press electrochemical flow reactor. The use of a turbulence promoter unexpectedly reduced the kinetics of TMX degradation (from 0.055 to 0.045 min^–1^), revealing a strong dependence of process performance on the flow configuration. Finally, the application of a modulated current strategy, adjusted to the instantaneous limiting current, substantially enhanced the kinetics of TMX degradation (0.055 vs 0.171 min^–1^), while significantly reducing energy consumption (25.5 vs 9.8 kWh m^–3^). Overall, the integration of laser-assisted synthesis, rational hydrodynamic design, and modulated current operation resulted in a robust and energy-efficient electrooxidation platform with clear potential for application in advanced water treatment systems.

## Graphical abstract

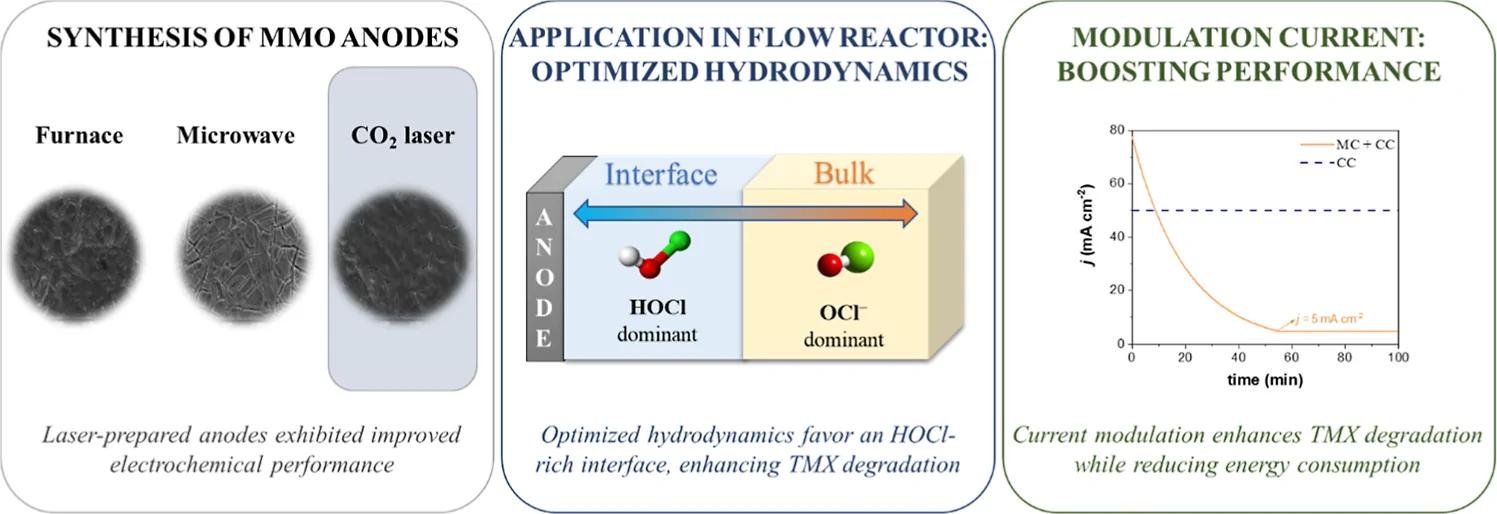

## 1 Introduction

Mixed metal oxide (MMO) anodes have emerged as highly promising materials for electrochemical advanced oxidation processes (EAOPs), particularly due to their stability, catalytic activity, and tunable surface properties.^1,2^ Relative to high-cost materials such as boron-doped diamond (BDD), MMO coatings can provide a more economically viable option, while maintaining practical activity for the transformation of a broad range of emerging organic contaminants.^3,4^

However, the electrochemical behavior of MMO coatings is strongly dependent on the thermal method used during their synthesis. Thermal treatment governs oxide phase development, crystallinity, and the spatial distribution and accessibility of active sites, and it can also influence coating adhesion and the long-term mechanical integrity of the catalytic layer. For this reason, nonconventional heating methods, such as laser irradiation and microwave-assisted calcination, have been explored as effective strategies to tailor MMO microstructure and performance.^5–8^

Several reports have indicated that MMO anodes prepared using rapid heating techniques can exhibit improved electrochemical characteristics, such as higher electrochemically active surface area and lower charge-transfer resistance, which are commonly associated with more favorable interfacial kinetics. These changes have been linked to enhanced electrocatalytic performance in the generation of chlorine-based oxidants from dilute chloride electrolytes.^8^ In particular, Ru-based MMO anodes produced by laser or microwave routes have been reported to favor hypochlorite and chloramine formation under relevant conditions, highlighting the sensitivity of oxidant speciation to synthesis-dependent surface structure and chemistry.^9^

In addition to oxidant generation, recent studies have highlighted that the anode material significantly influences not only the removal of target contaminants, but also the overall mineralization and the nature of the resulting transformation products. For instance, Hayat et al.^10^ demonstrated distinct degradation pathways during levofloxacin degradation using BDD and Ti/RuO_2_ anodes, which were associated with differences in the amount and nature of electrogenerated oxidants. At higher current densities, Ti/RuO_2_ generated larger amounts of oxidants, predominantly free chlorine, resulting in faster contaminant removal through mediated oxidation.^10^ The suitability of MMO anodes for environmental applications has been further confirmed by Herraiz-Carboné et al.^11^ during the electrochemical treatment of organochlorine-contaminated groundwater, underscoring the influence of operating conditions on degradation pathways.

Beyond electrode nature, Dória et al.^8^ demonstrated that the heating method used to prepare Ti/RuO_2_–TiO_2_ anodes strongly influenced their electrochemical performance, with the laser-optimized electrode exhibiting enhanced degradation and mineralization of organic contaminants during wastewater treatment, achieving higher removal efficiencies, extended electrode lifetimes, and reduced energy consumption, compared to conventionally prepared electrodes. These findings demonstrate the critical role of synthesis heating method in tailoring MMO anodes for environmentally relevant electrochemical applications. Therefore, the optimization of MMO anodes should consider not only their intrinsic electrochemical activity, but also their interaction with the operating conditions and reactor hydrodynamics.

Despite these advances, most synthesis-performance relationships for MMO anodes have been established under batch conditions and are often interpreted without explicitly accounting for the mass transport regimes that predominate in continuous-flow operation. Consequently, there is a practical technological gap in understanding the way that nonconventional heating during synthesis can influence performance under flow conditions, and whether the benefits observed in electrochemical metrics under static or batch operation persist when hydrodynamics and transport limitations govern oxidant production and pollutant conversion. Furthermore, electrode design and reactor operation are frequently optimized separately, even though the effective oxidizing environment at the electrode–electrolyte interface is jointly determined by surface properties and transport. Approaches such as increasing flow-induced convection and applying current modulation have been shown to promote renewal of the diffusion layer, improve the availability of reactive species at the electrode interface, and enhance oxidation and mineralization efficiencies.^12,13^ However, although current modulation has been explored as a strategy to improve the energy efficiency of electrochemical processes, its experimental implementation during electrolysis has received limited attention in chloride-containing media. In particular, the combination of experimentally implemented current modulation with Ru-based MMO anodes for the treatment of organic contaminants in chloride-containing electrolytes remained unexplored.

The hypothesis adopted in this work was that tailoring the MMO microstructure using laser irradiation and hybrid microwave heating could improve the density and accessibility of electroactive sites, consequently improving interfacial kinetics, while also enhancing coating durability. These synthesis-related advantages were expected to be most effectively expressed under flow operation when coupled with strategies to mitigate transport limitations (such as enhanced convection and current modulation). Accordingly, hydrodynamic optimization was considered not as an isolated reactor parameter, but as a complementary strategy to exploit the electrochemical properties of the synthesized anodes. Furthermore, because the current required for efficient pollutant oxidation changes as the treatment proceeds and mass-transfer limitations evolve, current modulation was investigated as a dynamic operational strategy to improve the charge utilization and reduce energy consumption.

To test this hypothesis, durable Ti/RuO_2_–Sb_2_O_4_–TiO_2_ anodes were synthesized using laser irradiation and hybrid microwave heating and compared with an electrode prepared by conventional furnace calcination. The electrodes were evaluated considering their structural properties, electrochemical behavior, and stability. The best-performing electrode was then scaled-up and evaluated for degradation of TMX using a flow reactor under different hydrodynamic conditions, allowing to identify operational parameters that could maximize treatment efficacy. Under the optimized hydrodynamic conditions, current modulation was applied as an additional operational strategy to reduce energy consumption. Thus, the present study integrates synthesis-tailored electrode design, hydrodynamic optimization, and dynamic current control within a single flow electrochemical treatment strategy. This integrated approach provide insight into how the synthesis route and reactor operation jointly govern MMO anode performance under realistic flow conditions, while addressing electrode durability, treatment efficiency, and energy consumption.

## 2 Experimental Section

### 2.1 Preparation of the MMO Anodes

Mixed metal oxide anodes with the nominal composition Ti/(RuO_2_)_0.8_–(Sb_2_O_4_)_0.1_–(TiO_2_)_0.1_ were synthesized using an ionic liquid route, as described previously.^9,14,15^ Briefly, ruthenium­(III) chloride (99%, Sigma-Aldrich), antimony­(III) chloride (99%, Sigma-Aldrich), and titanium­(IV) butoxide (97%, Sigma-Aldrich) were used as the metal precursors. These compounds were added in molar concentrations of 0.8 mol L^–1^ Ru, 0.1 mol L^–1^ Sb, and 0.1 mol L^–1^ Ti. The reagents were dissolved in the laboratory-prepared ionic liquid 2-hydroxyethylammonium acetate (2HEAA), under continuous stirring and heating at approximately 90 °C, until a homogeneous precursor solution was obtained.^16^ The precursor mixture was then applied to pretreated Ti plates, using a brush.

The coated substrates were calcined to thermally decompose the precursors and form the corresponding metal oxides. Three different heating methods were investigated: (i) thermal decomposition in a conventional furnace (model 3000 3P-S, EDG); (ii) localized heating using a CO_2_ laser (model GEM-100L, Coherent); and (iii) microwave-assisted heating (Consul microwave oven, 20 L capacity, maximum power of 700 W, frequency of 2.45 GHz). For all the methods, the final calcination temperature was 400 °C.

In the CO_2_ laser treatment, the coated substrates were exposed to a continuous laser beam that provided rapid and localized heating of the surface. The irradiation was applied in a controlled manner, ensuring a uniform thermal treatment of the coating and effective decomposition of the precursors.^7,17^ The hybrid microwave process employed a domestic microwave oven. Efficient heat retention was provided using an insulating chamber composed of refractory bricks, with SiC plates placed inside the cavity to act as microwave susceptors, enhancing microwave absorption and promoting uniform heating.^6,18^ For all the preparation methods, the coating and calcination steps were sequentially repeated until achieving a total oxide loading of 1.2 mg cm^–2^.

### 2.2 Structural and Morphological Characterizations

The crystalline phases in the MMO anodes were examined by X-ray diffraction (XRD), using a Bruker D8 ADVANCE ECO diffractometer operating with Cu Kα radiation, at 40 kV and 25 mA. The diffractograms were acquired between 20° and 80°, at 2° min^–1^, and were analyzed using X’Pert HighScore Plus software. The observed diffraction peaks were then matched with standard reference data from the International Centre for Diffraction Data (ICDD) database, to determine the crystalline oxide phases present in the samples.

The surface features of the anodes were evaluated by field-emission scanning electron microscopy (SEM) (GeminiSEM 500, Zeiss), with images acquired at magnifications of approximately 1000× and 5000×. The elemental distributions and qualitative chemical compositions of the coatings were obtained by energy-dispersive X-ray spectroscopy (EDX) integrated into the SEM system.

### 2.3 Electrochemical Characterizations

The electrochemical behaviors of the MMO electrodes were evaluated using a conventional three-electrode glass cell. The working electrode was Ti/(RuO_2_)_0.8_–(Sb_2_O_4_)_0.1_–(TiO_2_)_0.1_ with geometric area of 2 cm^2^. The reference electrode was Ag/AgCl and the counter electrode was a platinum plate (2 cm^2^ exposed area). All the measurements employed an Autolab PGSTAT302N potentiostat (Metrohm) and 80 mL of electrolyte prepared with ultrapure water (Milli-Q, resistivity >18 MΩ·cm, Millipore). All electrochemical characterization experiments were performed in triplicate using independently prepared electrodes.

Cyclic voltammetry (CV) measurements were performed between 0.2 and 1.2 V, at a scan rate of 50 mV s^–1^. Linear sweep voltammetry was performed in the potential range from 0.2 to 1.3 V, at a scan rate of 10 mV s^–1^. The morphology factors of the electrodes were estimated according to the procedure reported by Da Silva et al.^19^ Both voltammetric experiments were carried out in 3.7 g L^–1^ (0.06 mol L^–1^) NaCl (≥99.0%, Sigma-Aldrich).

Electrochemical impedance spectroscopy (EIS) was performed in 0.5 mol L^–1^ sulfuric acid solution (H_2_SO_4_, 95.0–98.0%, Sigma-Aldrich), applying a 5 mV perturbation amplitude over the frequency range 0.1–100 Hz. Accelerated service lifetime (ASL) experiments were performed in 0.5 mol L^–1^ H_2_SO_4_ solution, applying a current density of 500 mA cm^–2^, with the experiment being continued until the cell voltage reached 10 V, which was taken as the criterion for anode deactivation.

The electrode electroactive area was determined by cyclic voltammetry, using a redox probe solution containing 1.0 mmol L^–1^ potassium ferricyanide­(III) (K_3_[Fe­(CN)_6_], 99.0%, Sigma-Aldrich) and 0.1 mol L^–1^ potassium chloride (KCl, 99.0–100.5%, Sigma-Aldrich) as the supporting electrolyte. The electroactive area was calculated from the anodic peak current, using the Randles-Ševčík equation (eq 1)­

1 $$i_{p}=2.69\times 10^{5}n^{3/2}A_{E}D^{1/2}C\upsilon^{1/2}$$

where, *i*_p_ is the peak current (A), *n* is the number of electrons transferred (*n* = 1), *D* is the diffusion coefficient of ferricyanide (7.6 × 10^–6^ cm^2^ s^–1^), *C* is the K_3_[Fe­(CN)_6_] concentration (mol cm^–3^), and ν is the scan rate (V s^–1^).

### 2.4 Electrochemical Oxidation

A commercial formulation of thiamethoxan (TMX, Poncho brand) was employed for electrooxidation treatment using a bench-scale system equipped with the as-prepared MMO anode. The commercial formulation contains 600 g L^–1^ (60.0% w/v) of TMX as the active ingredient and 660 g L^–1^ of proprietary formulation ingredients, whose chemical composition is not disclosed by the manufacturer. The TMX concentration was quantified by HPLC using a PESTANAL analytical standard (Sigma-Aldrich) to prepare the external calibration curve. The TMX concentration in the degradation experiments was adjusted to 22 mg L^–1^ and the supporting electrolyte was a solution of NaCl (3.7 g L^–1^).

The experiments were performed using the recirculation system shown schematically in Figure 1a, with an electrolyte volume of 1.4 L. The electrochemical flow reactor (Figure 1b) was assembled using three acrylic plates. The central plate (5 mm thickness) defined the flow channel, while stainless steel (AISI 316) plates (6.0 × 10 cm) positioned on the outer sides acted as cathodes. The Ti/RuO_2_–Sb_2_O_4_–TiO_2_ anode (4.0 × 7.0 cm), synthesized using CO_2_ laser irradiation, was inserted into the central compartment. The resulting flow channel had a cross-sectional area of 2.5 cm^2^. The reactor was assembled using bolts and nuts and was sealed with O-rings to prevent leakage.

**Figure 1:**
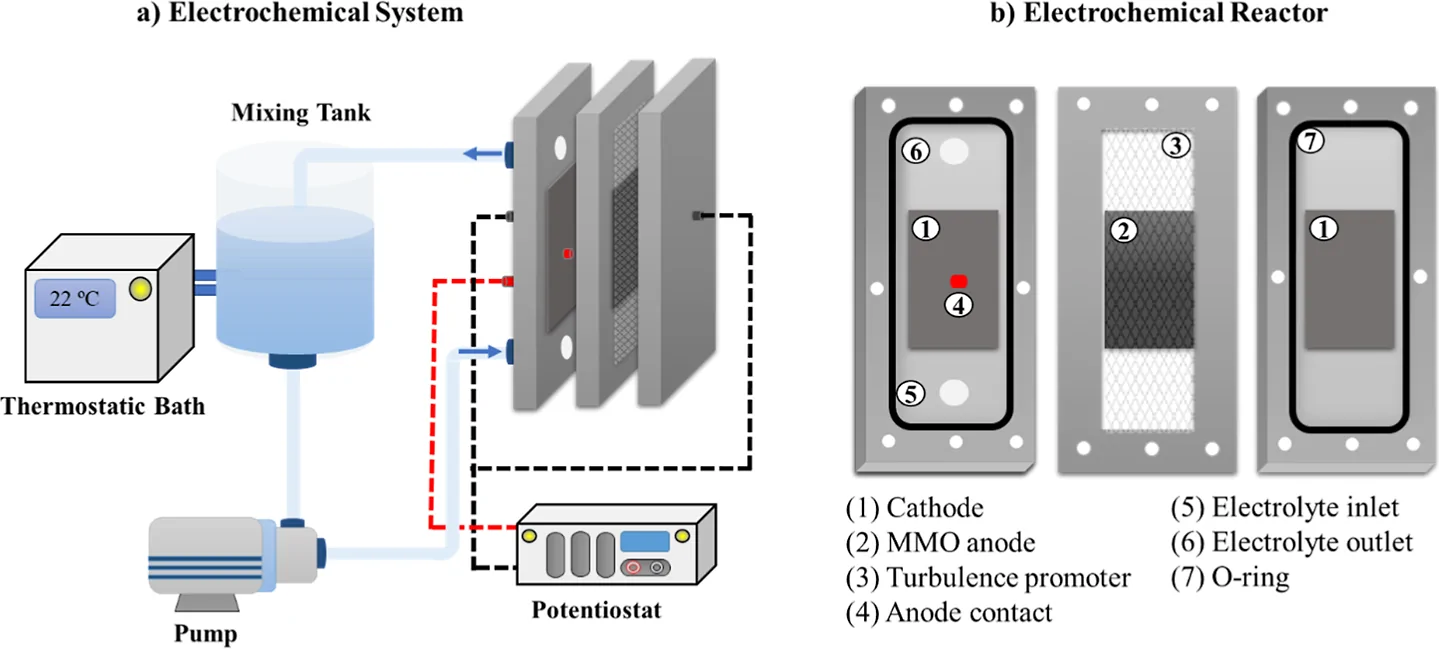
Schematic illustrations of (a) the experimental setup and (b) the electrochemical flow reactor.

First, TMX electrooxidation was evaluated under galvanostatic conditions (constant current, CC), to determine the optimal hydrodynamic regime. Flow velocities (*u*) of 0.13, 0.23, 0.30, and 0.37 m s^–1^ were investigated using the reactor without any turbulence promoter. A current density of 50 mA cm^–2^ was applied, calculated based on the geometric area of the electrode (*A*_G_ = 56 cm^2^), to ensure operation under mass transport control. All other experimental parameters were kept constant.

After defining the optimal flow conditions, the effect of a turbulence promoter was assessed by inserting a plastic mesh (4.0 × 7.0 cm, 1.0 mm thickness) into the flow channel between the cathode and the MMO anode, to enhance mixing and mass transport. Subsequently, the influence of pH was investigated. First, the pH was not controlled (initial pH ∼ 9) and was only monitored throughout the process. Additional experiments were then conducted under controlled conditions, at pH 4, to determine the effect on TMX degradation.

Aliquots were withdrawn at predetermined time intervals for analyses of chemical oxygen demand (COD), total organic carbon (TOC), and TMX concentration. All electrooxidation experiments were performed in duplicate using independent electrolysis runs, and the reported results correspond to the mean values of the replicate experiments.

### 2.5 Electrochemical Oxidation Using the Modulated Current Method

Since the electrooxidation process was performed using an active anode, TMX removal was expected to proceed primarily by indirect oxidation mediated by electrogenerated active chlorine species. Under such conditions, the overall oxidation rate may be controlled by the mass transport of chloride ions from the bulk solution to the electrode surface, where chloride is oxidized to reactive chlorine species.

Accordingly, the mass transfer coefficient, *k*_m,Cl^–^_, was determined by considering chloride ions as the electroactive species governing the process, rather than the target organic compound. The *k*_m,Cl^–^_ value was estimated based on the film diffusion model, assuming diffusion-limited mass transport across the hydrodynamic boundary layer, described by eq 2. The diffusion coefficient of chloride (*D*_Cl^–^_) in aqueous solution was taken as 2.03 × 10^–9^ m^2^ s^–1^,^20,21^ while the diffusion layer thickness (δ) was determined experimentally (see in the Text S1 of the Supporting Information for details).

2 $$k_{m,{Cl}^{-}}=\frac{D_{{Cl}^{-}}}{\delta }$$

Using the obtained *k*_m_ value, the limiting current density (*i*_lim_) was estimated based on the bulk chloride concentration, according to eq 3, where *n* is the number of electrons involved in the reaction and *F* is the Faraday constant.

3 $$i_{\lim}\left(t\right)=nFk_{m,{Cl}^{-}}AC_{{Cl}^{-}}\left(t\right)$$

The modulated current strategy was implemented by adjusting the applied current to remain close to the instantaneous limiting current (*i*_appl_(*t*) = *i*_lim_(*t*)). The time-dependent applied current was calculated based on the mass transfer-controlled model for the limiting current (eq 4). Current modulation was maintained until the current density reached a minimum value of 5 mA cm^–2^. Below this threshold, electrolysis was continued under constant current conditions, resulting in a combined modulated current and constant current operation (MC + CC). During the modulation step, the applied current was decreased stepwise at 30 s intervals, which was established based on the findings of Oliveira et al.,^22^ who reported that shorter intervals did not lead to improved process performance.

4 $$i_{app}\left(t\right)=nFk_{m}AC_{{Cl}^{-},0}\;\exp\left(-\frac{k_{m}A}{V}t\right)$$

This approach enabled estimation of the time-dependent chloride-controlled current profile by considering the progressive decrease in chloride availability for anodic chlorine-derived oxidant generation.

### 2.6 Analytical Procedures

Aliquots (2.0 mL) of the sample were transferred to commercial COD digestion vials (LR range, 3–150 mg L^–1^, Hach) and digested at 150 °C for 2 h in a thermoreactor (DBR 200, Hach), according to the manufacturer’s instructions. After cooling to room temperature, the absorbance was measured at 600 nm using a colorimeter (DR 890, Hach). TOC was quantified using a Sievers InnovOx 900 analyzer (GE Analytical Instruments), based on chemical oxidation with sodium persulfate (Na_2_S_2_O_8_, 30% w/v, ≥98%, Sigma-Aldrich) in a phosphoric acid medium (H_3_PO_4_, 6.0 mol L^–1^, 85%, Panreac).

The TMX concentration was determined by high performance liquid chromatography (HPLC), using a Shimadzu system equipped with a reverse-phase Ascentis Express C18 column (100 × 4.6 mm, 2.7 μm particle size). The mobile phase consisted of water and methanol (CH_3_OH, ≥99.9%, Sigma-Aldrich) at a ratio of 75:25 (v/v), delivered isocratically at a flow rate of 0.5 mL min^–1^. The injection volume was 20 μL and the column temperature was 35 °C. A UV detector was set at 251 nm. These conditions resulted in a TMX retention time of approximately 5.6 min. A TMX analytical standard (PESTANAL, Sigma-Aldrich) was used for construction of the calibration curve.

### 2.7 Electrooxidation Performance Metrics

The kinetics of TMX removal during the electrochemical process was evaluated assuming a pseudo-first order kinetic model, which is commonly applied to the electrochemical degradation of dilute organic contaminants occurring under mass transfer control.

The apparent pseudo-first order rate constant (*k*_app_, min^–1^) was determined from the linearized kinetic expression (eq 5), where *C*_0_ and *C* (mg L^–1^) are the TMX concentration at *t* = 0 and time *t* (min), respectively. The *k*_app_ value was obtained by linear regression of the experimental data. Pseudo-first order behavior is often consistent with electrochemical oxidation under conditions where the overall rate is governed by mass transport and/or an approximately constant steady-state oxidant flux, and has been reported for the degradation of organic contaminants in aqueous media.^23,24^

5 $$\ln\left(\frac{C}{C_{0}}\right)=-k_{app}t$$

The energy performance of the electrochemical process was evaluated using two complementary metrics: the specific electrical energy consumption (EC) and the electrical energy per order (*E*_EO_). The combined use of EC and *E*_EO_ enables distinction between operational electrical demand and effective pollutant removal efficacy. The applied current (*I*, A), the electrolysis time (*t*, h), and the treated solution volume (*V*_s_, m^–3^). For CC operation, the EC was calculate based on the average cell voltage (*U*_cell_, V), while in MC operation, in which both the applied current and cell potential varied with time, the EC was calculated by integrating the instantaneous electrical power over the electrolysis time, according to equation $E=\int_{0}^{t}U\left(t\right)I\left(t\right)dt$ . The applied current was obtained from the calculated modulation profile, while the cell potential was obtained from the corresponding potential-versus-time profile.

The specific energy consumption, defined as the electrical energy input per unit volume of treated solution, was calculated according to eq 6. To account for the extent of contaminant removal, *E*_EO_ was calculated as the energy required to reduce the TMX concentration by 1 order of magnitude (90%), as expressed by eq 7.

6 $$EC\;\left(kWh{\;m}^{-3}\right)=\frac{U_{cell}It}{Vs}$$

7 $$E_{EO}\;\left(kWh\;m^{-3}{\;order}^{-1}\right)=\frac{U_{cell}It}{V\;\log\left(\frac{C_{0}}{C_{t}}\right)}$$

The current efficiency (CE) was used to determine the fraction of the applied current that was effectively involved in organic matter oxidation. This was calculated from the COD decay according to eq 8, where COD_0_ and COD_*t*_ are the initial and final COD values (mg O_2_ L^–1^), *F* is the Faraday constant (96,487 C mol^–1^), *V* is the solution volume (L), *I* is the applied current (A), and *t* is the electrolysis time (s).

8 $$CE\;\left(\%\right)=\frac{\left({COD}_{0}-{COD}_{t}\right)FV}{8\int It}$$

## 3 Results and Discussion

### 3.1 Microstructural and Morphological Analysis

Figure 2 shows the XRD patterns of the ternary Ti/RuO_2_–Sb_2_O_4_–TiO_2_ anodes prepared using the furnace, microwave, and CO_2_ laser heat treatments. Identification of the diffraction peaks was by comparison with reference data from the ICDD database. The dominant reflections were consistent with rutile-type RuO_2_ (ICDD No. 40-1290), Sb_2_O_4_ (ICDD No. 01-071-0383), and TiO_2_ (ICDD No. 01-076-0320). For all samples, the patterns were largely governed by rutile-type reflections, indicating that the oxide coatings crystallized predominantly in a rutile-related framework.

**Figure 2:**
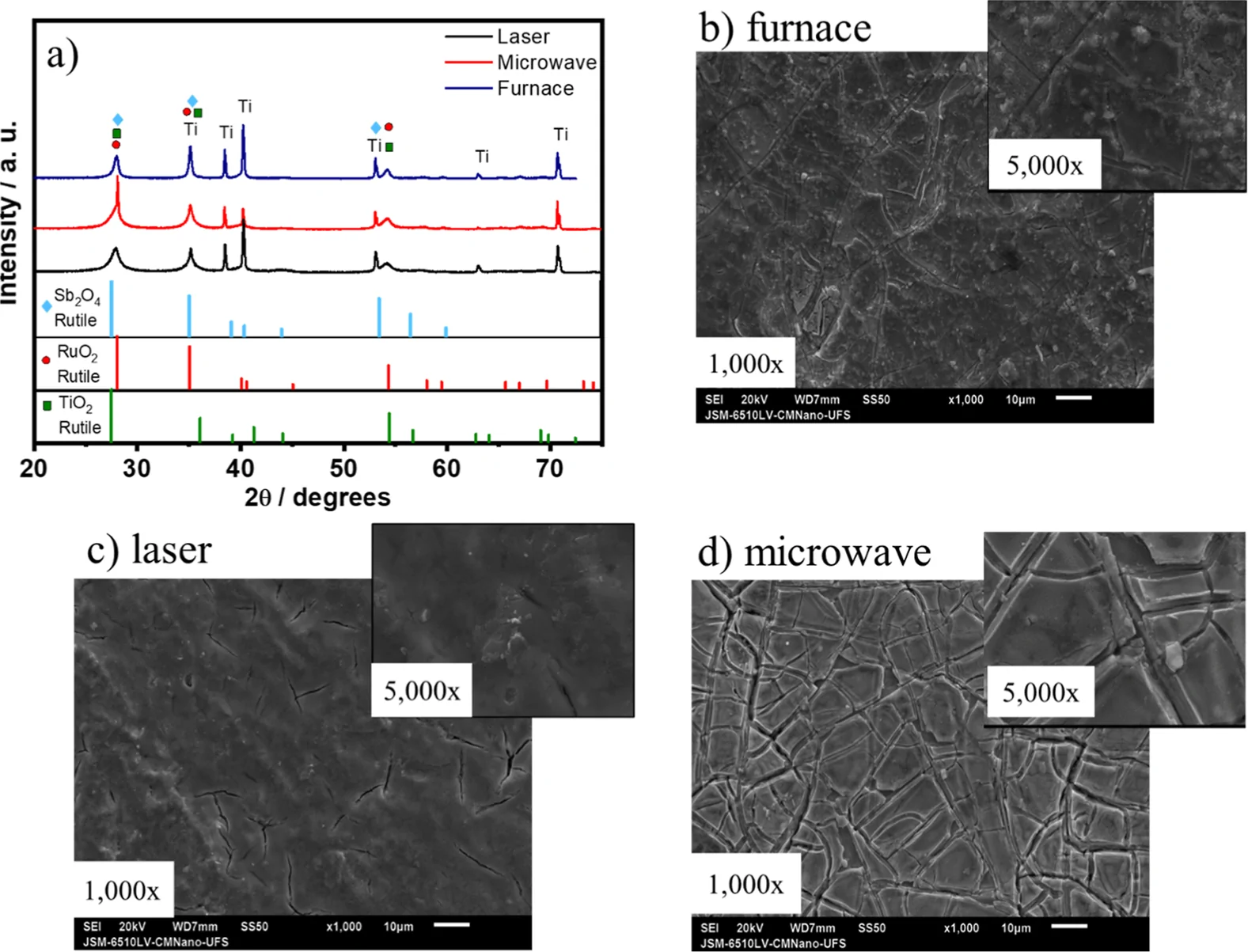
X-ray diffraction patterns (a) and SEM micrographs of the Ti/RuO_2_–Sb_2_O_4_–TiO_2_ anodes synthesized using the conventional furnace (b), laser (c), and microwave irradiation (d) heat treatment methods.

The substantial peak overlap among RuO_2_, TiO_2_, and Sb_2_O_4_ reflected their structural similarity and suggested the formation of a solid solution based on RuO_2_, with Ti and Sb species incorporated into the rutile lattice, particularly considering the high nominal Ru fraction (80 mol %). This type of structural behavior has been reported for binary RuO_2_–TiO_2_ and RuO_2_–Sb_2_O_4_ anodes, indicative of the incorporation of secondary metal oxides into the rutile lattice, leading to overlapping diffraction peaks and the formation of mixed rutile-type structures.^7–9,16^

Peaks associated with the Ti substrate (ICDD No. 44-1294) were present in all the diffraction patterns, confirming the contribution of the metallic support to the overall signal. Notably, these Ti-related peaks were more prominent for the anode prepared using conventional furnace calcination, suggesting lower effective coverage or a thinner and/or less uniform oxide layer. Similar features have been reported for MMO anodes prepared by different routes, with increased Ti peak intensity being associated with coating discontinuities and incomplete surface coverage.^7–9^ In the present work, the lower intensity of Ti reflections for the anodes prepared using the microwave and especially the laser treatments was consistent with improved continuity of the coating and a lower fraction of exposed substrate, in agreement with previous work using nonconventional heating methods.^9^

Greater exposure of the Ti substrate can compromise electrochemical durability, because uncovered regions facilitate access of the electrolyte to the substrate, leading to localized corrosion and accelerated passivation under anodic polarization, potentially resulting in premature performance loss. The lower intensity of Ti-related reflections for the microwave- and laser-treated anodes suggested more effective surface coverage, which would be expected to improve operational stability. These features showed that continuous MMO layers can delay deactivation by limiting the penetration of electrolyte to the Ti support and by reducing the mechanical and chemical stresses associated with gas evolution during anodic operation.^25,26^

The SEM images at 1000× magnification of the anodes prepared using the different heating methods (Figure 2b–d) revealed rough surfaces for all the samples, with the typical cracked-mud morphology observed for MMO coatings prepared by thermal decomposition techniques.^5,7–9^ Nevertheless, there were significant differences in surface morphology, according to the type of thermal treatment applied.

The electrode subjected to conventional furnace treatment (Figure 2b) presented a heterogeneous surface morphology characterized by poorly defined fissures and a nonuniform distribution of the catalytic coating. In contrast, the laser-treated electrode (Figure 2c) showed a denser and more homogeneous morphology, with a lower crack density and an apparently smoother surface. The cracks were more widely spaced and shallower, suggesting that the rapid and localized heating acted to improve adhesion of the film to the substrate, while mitigating excessive thermal stresses during coating formation. The microwave-treated electrode (Figure 2d) exhibited a more highly cracked morphology, with well-defined and interconnected fissures, indicating the formation of a thicker and more rigidly adhered film. This could be attributed to the high thermal gradients and volumetric heating characteristic of microwave processing, which intensified internal stresses during calcination.

These results confirmed that the type of heating methodology can strongly affect the pore size distribution and compactness of coatings on MMO anodes. Dória et al.^8^ reported that electrodes prepared using conventional furnace treatment presented broader pore size distributions and larger pores, while anodes synthesized using laser or microwave heating displayed narrower pore size distributions and significantly smaller pores, accompanied by shallower surface cracks. Notably, laser-treated coatings appeared more compact, a characteristic associated with improved surface recovery and potentially enhanced electrode lifetime.

### 3.2 Electrochemical Characterization

Figure 3a shows cyclic voltammograms recorded with the anodes prepared using the different thermal treatments, in the potential window 0.2–1.2 V vs Ag/AgCl, in 3.7 g L^–1^ NaCl electrolyte. All the currents were normalized to the geometric area of the working electrode. The voltammetric profiles exhibited a quasi-rectangular shape, indicating predominantly capacitive behavior, characteristic of mixed metal oxide anodes.^6^ No well-defined reversible redox peaks associated with Ru-oxidation–reduction transitions were observed. This behavior was as expected for measurements in chloride media, where the lack of proton participation suppresses proton-coupled Ru redox processes, resulting in a voltammetric signature dominated by nonfaradaic double-layer charging.^9,27^

**Figure 3:**
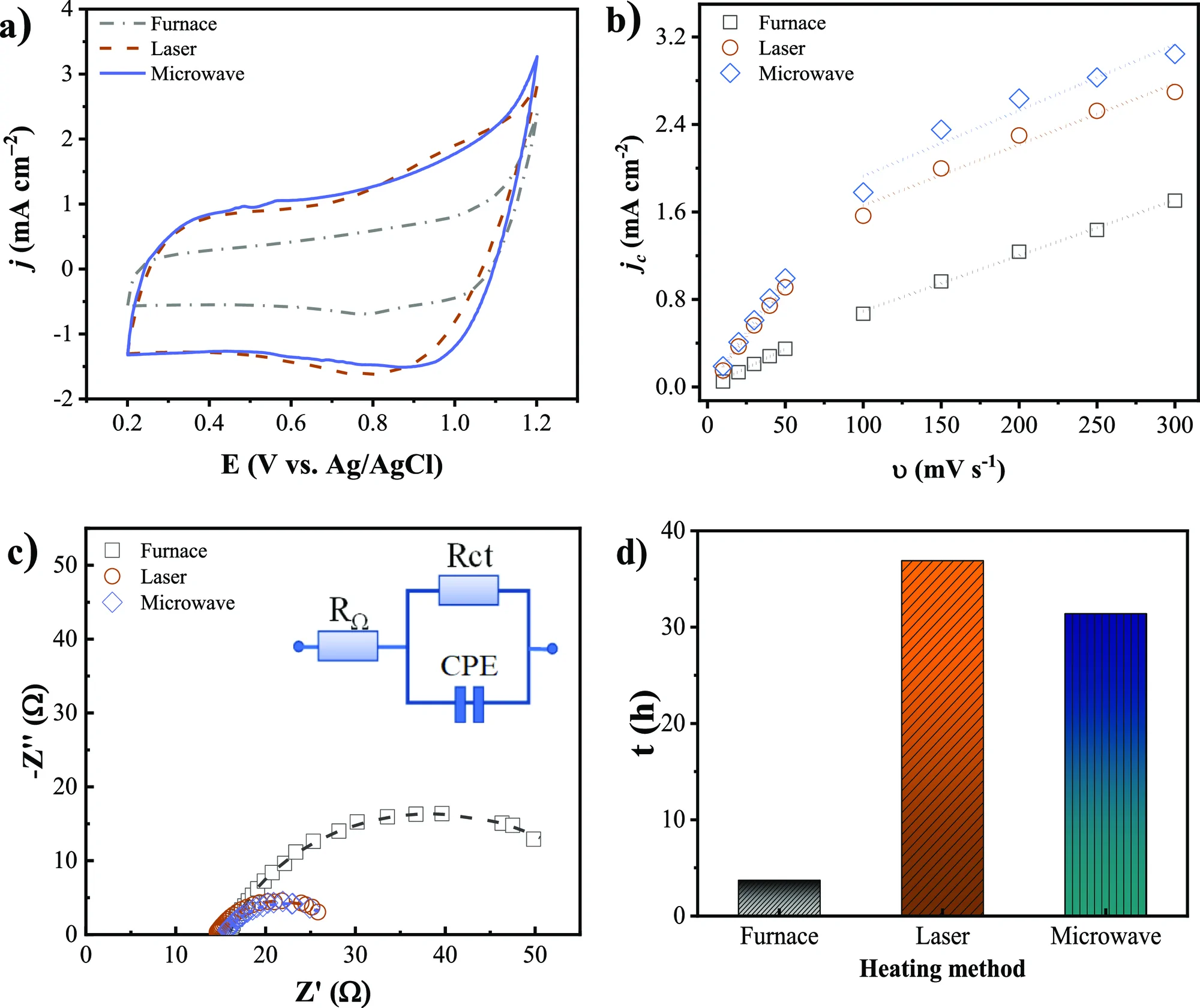
(a) Cyclic voltammograms, (b) dependence of the capacitive current density, *j*_c_, on the potential sweep rate, (c) Nyquist impedance plots, and (d) accelerated service lifetime (ASL) results for the Ti/RuO_2_–Sb_2_O_4_–TiO_2_ anodes prepared using the different thermal treatments. (a) and (b) Were recorded in 3.7 g L^–1^ NaCl, whereas (c) and (d) were obtained in 0.5 mol L^–1^ H_2_SO_4_. The ASL test was performed at 500 mA cm^–2^.

The electroactive surface area (*A*_E_) was determined according to eq 1, using the slope of the linear fit of the plot of *i*_p_ vs *v* ^1/2^ (Figure S4d and Table S1), where *i*_p_ denotes the anodic peak currents extracted from successive cyclic voltammograms (Figure S4a–c). For all the electrodes, the *A*_E_ value exceeded the *A*_G_ of 2 cm^2^, increases to 5.29, 10.28, and 10.72 cm^2^ for the electrodes treated using the furnace, microwave, and laser methods, respectively. These results indicated that nonconventional heating routes can favor the development of an electrochemically accessible surface area, relative to conventional furnace treatment, in agreement with the morphological features observed by SEM.

Dória et al.^9^ investigated similar calcination strategies for the preparation of binary Ru-based anodes. It was observed that Ti/RuO_2_–Sb_2_O_4_ electrodes synthesized using nonconventional heating methods (microwave and laser) presented voltammetric charges (a relative measure of electroactive surface area) 1.9 and 1.4 times higher, respectively, compared to the use of conventional furnace treatment. Similarly, for Ti/RuO_2_–TiO_2_ electrodes, microwave and laser processing resulted in voltammetric charge increases of 2.7 and 2.9 times, respectively, compared to the furnace method. These results reinforce the ability of nonconventional thermal routes to significantly increase the electroactive surface area of electrodes.

As shown in Figure 3b, for all the electrodes, the capacitive current density (*j*_c_) increased linearly with the scan rate (ν). This linear dependence indicated that charge storage was governed predominantly by capacitive processes, with a negligible contribution from diffusion-controlled faradaic reactions. Such behavior is characteristic of pseudocapacitive or surface-controlled systems, where charge compensation occurs by fast and reversible processes at (or near) the oxide surface, rather than by diffusion-limited redox reactions in the bulk medium.^28^

Separation of the external (*C*_e_) and internal (*C*_i_) capacitance contributions enables a more accurate assessment of the accessibility of electroactive sites on the anode surface. The total capacitance (*C*_t_) can be calculated as the sum of the internal and external contributions (*C*_t_ = *C*_i_ + *C*_e_). At low scan rates, electrochemical reactions occur at nearly all the active sites, whereas at high scan rates only the most readily accessible sites contribute to the response.^19,29^ Based on this behavior, the morphological factor (φ) can be calculated, defined as the ratio φ = *C*_i_/*C*_t_. Values of φ close to 1 indicate a predominance of internal sites, while values approaching zero reflect a dominant contribution of external sites.

All the anodes, irrespective of the synthesis route, showed a more pronounced contribution from internal sites (Table 1). The electrode obtained using the microwave method showed the highest values for the internal capacitance (16.90 mF cm^–2^) and the morphological factor (φ = 0.82), indicating a substantial contribution of internal porosity. This was closely followed by the electrode obtained by the laser method (φ = 0.71), while the furnace-treated electrode presented the lowest φ value (0.57), consistent with a less developed morphology and limited utilization of the available surface area.

**Table 1:** Electrochemical Properties and Summary of Fitted EIS Data for the Ti/RuO_2_–Sb_2_O_4_–TiO_2_ Anodes Synthesized Using the Furnace, Laser, and Microwave Heating Procedures

	heating method		
parameter	furnace	laser	microwave
*A*_E_ (cm^2^)	5.29	10.72	10.28
*C*_t_ (mF cm^–2^)	7.60	18.80	20.60
*C*_e_ (mF cm^–2^)	3.30	5.40	3.70
*C*_i_ (mF cm^–2^)	4.30	13.40	16.90
φ	0.571 ± 0.09	0.711 ± 0.02	0.817 ± 0.09
*E* (V vs Ag/AgCl)	1.15	1.15	1.15
*R*_Ω_ (ohm)	15.95	14.56	15.58
*R*_ct_ (ohm)	44.16	13.95	12.54
CPE (F)	0.03	0.08	0.06
*n*	0.82	0.74	0.77
χ^2^ × 10^3^	2.88	1.16	3.29

The Nyquist plots obtained from the EIS measurements are shown in Figure 3c. These measurements were performed in the initial region of the oxygen evolution reaction (OER), determined previously from the linear sweep voltammetry (LSV) measurements shown in Figure S5. The EIS spectra exhibited depressed semicircles, characteristic of mixed metal oxide anodes.

The experimental data were fitted using a Randles-type equivalent circuit (inset in Figure 3c), comprising a solution resistance (*R*_s_) in series with a parallel combination of the charge-transfer resistance (*R*_ct_) and a constant phase element (CPE). The fitted parameters are summarized in Table 1. *R*_s_ accounts for the ohmic resistance of the electrolyte, coating, and electrical contacts, while *R*_ct_ is associated with the charge-transfer process of the OER and correlates with the diameter of the semicircles. The CPE describes the nonideal capacitive behavior of the electrodes, reflecting the effects of surface roughness, porosity, coating composition, and nonuniform distributions of potential and current. The CPE exponent (*n*) ranges from 0 to 1, where *n* = 1 corresponds to ideal capacitive behavior.^30,31^

The charge-transfer resistance values (Table 1) indicated significantly lower resistance of the anodes synthesized using the nonconventional methods, compared to the furnace-prepared electrode. The *R*_ct_ values for the electrodes obtained using the microwave and laser methods were 12.54 and 13.95 Ω, respectively, approximately three times lower than the *R*_ct_ for the furnace-prepared electrode (44.16 Ω). These results were indicative of more efficient charge transfer kinetics for the electrodes prepared using the rapid heating methods.

The service lifetime of anodes is a critical parameter for industrial applications. Therefore, ASL tests were performed to evaluate the influence of the heating method on the stability of the ternary anodes (Figure 3d). Under harsh operating conditions (500 mA cm^–2^ in 0.5 mol L^–1^ H_2_SO_4_), the laser-prepared anode had the longest lifetime of approximately 37 h, followed by the microwave-treated anode (around 31 h). In contrast, the anode obtained using the conventional furnace method showed a significantly shorter lifetime of approximately 4 h. These results demonstrated that rapid heating methods can not only enhance electrochemical activity, but also improve the structural stability of the MMO coating under severe anodic polarization.

In previous work, use of the laser heating method for anode synthesis significantly increased the service lifetime of Ti/RuO_2_–TiO_2_ anodes, achieving improvements of up to 1.94 and 4.45 times, compared to electrodes prepared using microwave and furnace heating, respectively.^8^ Notably, in this previous study it is show that the laser-prepared electrode exhibited a longer service lifetime than the microwave-prepared electrode, supporting the influence of coating morphology on the stability observed in the present work. The enhanced stability was attributed to differences in coating morphology and degradation mechanisms. The deactivation of Ru-based anodes was mainly related to oxide layer dissolution and the formation of nonconductive passive films, which are processes strongly influenced by the synthesis method.^8,32^

Compared to other reported Ru-based MMO electrodes evaluated under identical accelerated service lifetime conditions, the stability achieved in the present study underscores the competitiveness of the proposed anode. Santos et al.^16^ reported a maximum service lifetime of 22 h lifetime for Ti/(RuO_2_)_0.8_–(Sb_2_O_4_)_0.2_ anodes prepared using 2HEAA and conventional furnace. Similarly, Dória et al.^8^ reported a service lifetime of approximately 20 h for Ti/RuO_2_–TiO_2_ anodes prepared by Pechini method and laser heating. More recently, Castro et al.^33^ reported an accelerated service lifetime of approximately 35 h for Ti/(RuO_2_)_0.8_–(Sb_2_O_4_)_0.2_ anodes prepared using dipropylammonium-based ionic liquids in furnace.

Coatings with more cracked structures (typically obtained by conventional furnace treatment) facilitate the penetration of water toward the metallic substrate, accelerating passivation and reducing the electrode lifetime. In contrast, more compact and homogeneous films, such as those obtained by laser heating, act as more effective barriers against electrolyte infiltration, thereby improving the durability of the anode, as observed here for the electrodes prepared using the nonconventional methods.

One of the main challenges in environmental electrochemistry is the development of anodes that combine high electrocatalytic activity, low cost, and, most importantly, long-term durability.^34^ In this work, CO_2_ laser heating proved to be an effective approach for improving the electrochemical properties of the electrodes, highlighting the significant increase in service lifetime. Therefore, given its superior performance, the laser-treated electrode was scaled up from 2 to 56 cm^2^, with evaluation of its catalytic efficiency in a flow reactor. An additional advantage of the CO_2_ laser-assisted synthesis is its scalability. Since the laser beam is scanned over the Ti substrate using a computer-controlled translation stage, the preparation of larger electrodes can be readily achieved by extending the scanning path without modifying the synthesis protocol,^35^ highlighting the potential of this approach for the fabrication of larger MMO electrodes required for practical electrochemical reactor applications.

### 3.3 Electrooxidation of TMX

The electrooxidation of TMX was investigated using a flow-through electrochemical reactor operated at a constant current density of 50 mA cm^–2^. The influence of hydrodynamic conditions on TMX degradation kinetics and mineralization was assessed by varying the flow velocity (*u*) and by introducing a turbulence promoter (TP) to enhance mass transfer. The effect of pH was evaluated by comparing experiments conducted under uncontrolled conditions with those performed at controlled acidic pH (pH 4), selected to favor the formation and stability of active chlorine species (mainly HOCl) during the electrochemical process. In addition, TMX degradation under a modulated current (MC) regime was examined and compared with conventional galvanostatic operation.

#### 3.3.1 Effect of pH

The effect of pH on TMX degradation was investigated to elucidate the role of chlorine speciation in the electrochemical oxidation process. In chloride-containing electrolytes, oxidation using active anodes is widely accepted to proceed predominantly via mediated pathways involving electrochemically generated chlorine species, rather than direct electron transfer at the electrode surface.^7,36–38^ Under such conditions, the overall oxidation rate is closely related to mass transfer phenomena, particularly the transport of chloride ions toward the anode surface, as supported by the mass transfer analysis presented in the Supporting Information. Active chlorine species are generated at the anode surface by the oxidation of chloride ions, according to eqs 9–12.

9 $$2{Cl}^{-}\to {Cl}_{2}+2e^{-},\left(E^{{}^{\circ}}=1.36\;V\;vs\;SHE,\;{Cl}_{2}/{Cl}^{-}\right)$$

10 $${Cl}_{2}+H_{2}O⇌HOCl+H+{Cl}^{-},\left(E^{{}^{\circ}}=1.49\;V\;vs\;SHE,\;HOCl/{Cl}^{-}\right)$$

11 $$HOCl⇌H^{+}+{OCl}^{-}$$

12 $${OCl}^{-}+H_{2}O+2e^{-}\to {Cl}^{-}+2{OH}^{-},\left(E^{{}^{\circ}}=0.89\;V\;vs\;SHE,\;{OCl}^{-}/{Cl}^{-}\right)$$

These reactions lead to the formation of chlorine, which undergoes hydrolysis to produce hypochlorous acid and hypochlorite, depending on the solution pH. According to well-established chlorine speciation equilibria, hypochlorous acid predominates under acidic conditions, while hypochlorite becomes dominant under alkaline conditions, as illustrated by the chlorine speciation diagram (Figure S3). This distinction is particularly relevant because hypochlorous acid exhibits a significantly higher oxidation potential than hypochlorite (approximately 1.49 and 0.89 V vs SHE, respectively). Therefore, the efficiency of oxidation of organic contaminants is strongly influenced by the prevailing chlorine species in solution.

To evaluate this effect, experiments were conducted under two representative conditions: (i) uncontrolled pH, corresponding to the natural pH of the solution (pH ∼ 9); and (ii) controlled acidic conditions (pH 4). The alkaline condition reflected the inherent pH of the commercial pesticide formulation, while the acidic condition was selected to favor the formation of more reactive chlorine species, according to equilibrium considerations.

The results showed that TMX degradation was significantly enhanced under acidic conditions (Figure 4), with the apparent kinetic constant increasing from 0.055 min^–1^ at pH 9 to 0.12 min^–1^ at pH 4, corresponding to an approximately 2-fold increase. This behavior is consistent with the higher oxidative strength associated with hypochlorous acid and supports the hypothesis that chlorine speciation contributes to the observed degradation behavior. Although the distribution of chlorine species was not directly quantified in this study, the observed trends were consistent with well-established chlorine speciation equilibria and the corresponding oxidation potentials reported in the literature. These findings demonstrated that the electrochemical oxidation process was strongly governed by oxidant speciation, providing a basis for interpreting the influence of hydrodynamic conditions, discussed in the next section.

**Figure 4:**
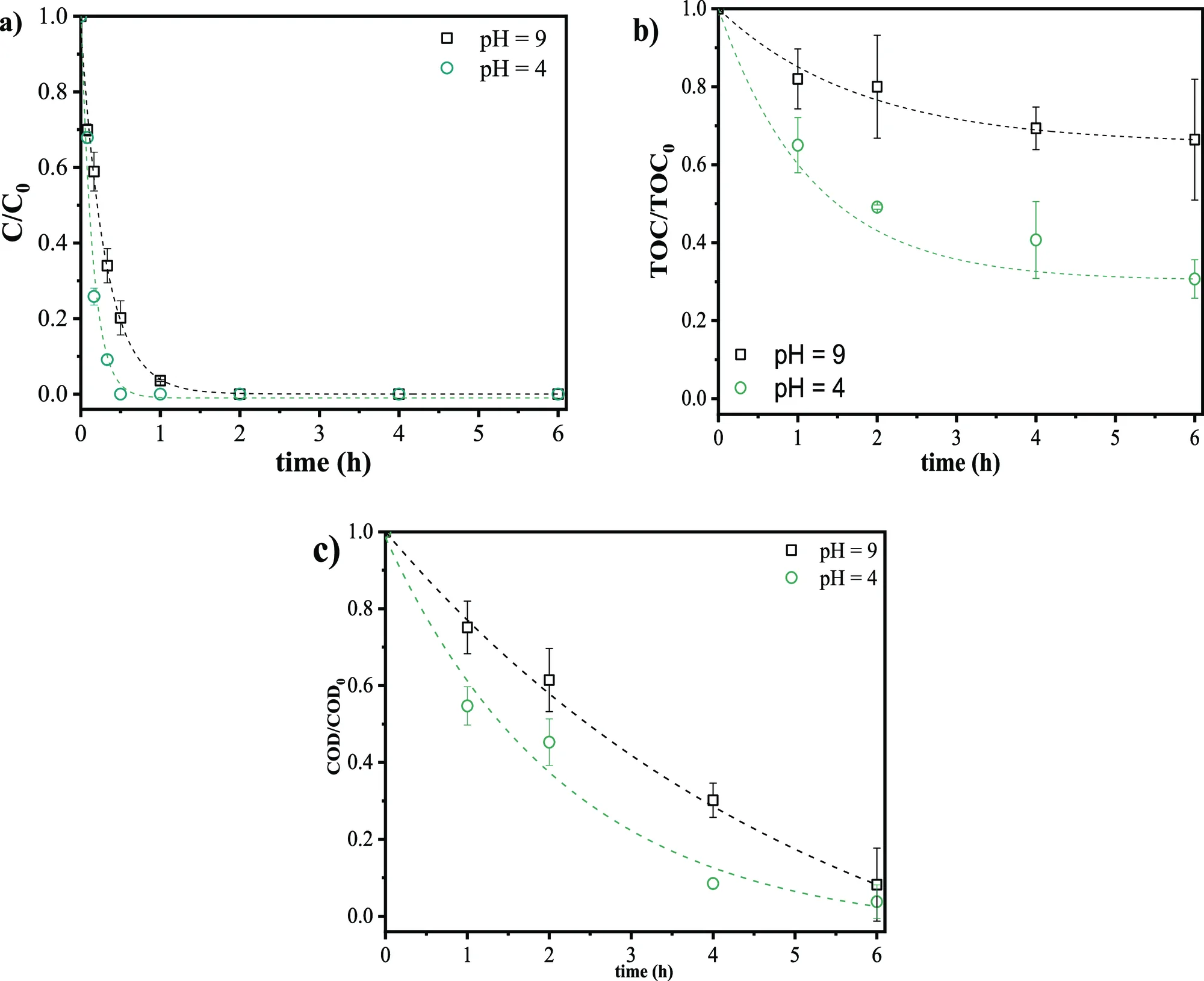
Normalized plots of TMX concentration (a), TOC (b), and COD (c), as a function of electrolysis time, at pH 4 and pH_0_ 9. Conditions: Ti/RuO_2_–Sb_2_O_4_–TiO_2_ anode synthesized using CO_2_ laser heating; COD_0_ = 100 mg O_2_ L^–1^; TOC_0_ = 28 mg L^–1^; pH = 9; *j* = 50 mA cm^–2^; *V* = 1.4 L; NaCl = 3.7 g L^–1^; *u* = 0.23 m s^–1^.

A similar trend was observed for mineralization. After 6 h of electrolysis, TOC removal reached 69.3% at pH 4, while only 33.6% removal was achieved at pH 9. The removal of COD exceeded 90% in both cases, indicating efficient oxidation of organic compounds, although acidic conditions favored greater mineralization.^39^ The current efficiency was calculated from the COD vs time curves, giving values of 25.6% and 26.9% at pH 9 and 4, respectively, indicating that at both pH values a significant part of the applied charge was diverted to parallel reactions, notably the OER.^5,40^

Despite the improved performance observed at pH 4, subsequent investigations were conducted at pH 9. This choice was based on practical considerations, since this condition corresponded to the natural pH of the solution, without the need for chemical adjustment. Operating under acidic conditions would require continuous acid addition to counteract the buffering capacity of the system, which would increase reagent consumption and operational costs. Therefore, pH 9 was selected as a more realistic and economically feasible condition for further studies.

#### 3.3.2 Effects of Flow Velocity and Turbulence Promoter

Figure 5 shows the normalized TMX concentration, TOC, and COD, as a function of time, for different flow velocities. Rapid degradation of TMX occurred under all the operating conditions, with removals exceeding 90% after 120 min of electrolysis, demonstrating the high electrocatalytic efficiency of the anode. The TMX concentration decay followed pseudo-first order kinetic behavior (eq 5), typical of processes controlled by mass transfer limitations and/or mediated electrochemical oxidation pathways.^35,41,42^

**Figure 5:**
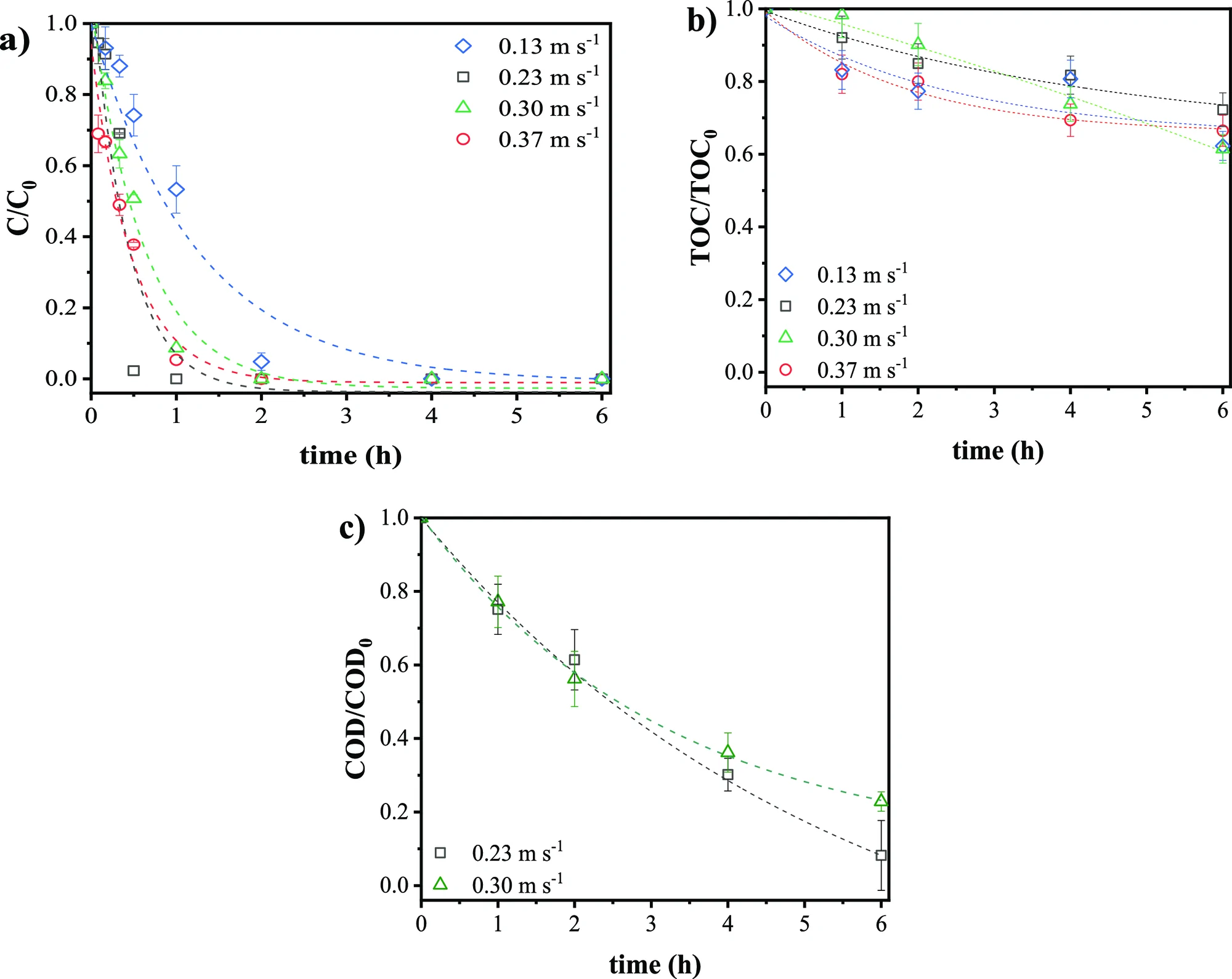
Normalized plots of TMX removal (a), TOC (b), and COD (c), as a function of time, for different flow velocities. Conditions: TMX_0_ = 22 mg L^–1^; COD_0_ = ∼100 mg L^–1^; TOC_0_ = 28 mg L^–1^; pH_0_ = 9.0; *j* = 50 mA cm^–2^; NaCl = 3.7 g L^–1^.

The flow velocity significantly affected the degradation kinetics, with the apparent rate constant increasing from 0.019 min^–1^ at 0.13 m s^–1^ to 0.055 min^–1^ at 0.23 m s^–1^, showing that a moderate increase of the flow rate could enhance process performance. During the experiments, the pH varied within a narrow range, between pH 8.5 and 9.55 (Figure S6), indicating that the differences in degradation behavior could not be attributed to altered pH of the bulk medium. Therefore, the observed behavior could be explained by hydrodynamic effects, especially enhanced mass transfer, which increased the flux of chloride ions toward the anode surface and favored the generation of oxidizing species.

However, a further increase of the flow velocity from 0.23 to 0.37 m s^–1^ resulted in a slow degradation rate, with the kinetic constant decreasing to 0.020 min^–1^, demonstrating that additional hydrodynamic turbulence did not necessarily improve the overall electrooxidation rate. Besides the effect on TMX degradation, the hydrodynamic conditions also influenced the mineralization behavior, with the highest TOC and COD removals obtained at the intermediate flow velocity of 0.23 m s^–1^, reaching approximately 49.0% and 91.8% removal, respectively, after 6 h of electrolysis. Higher flow velocities resulted in lower removals, indicating that the mineralization efficiency followed the same trend as the degradation kinetics.

The effect of the turbulence promoter was similar to that of the flow rate. Although installation of the mesh was expected to enhance mixing and reduce the thickness of the diffusion layer, the degradation kinetics decreased from 0.055 min^–1^ (without the promoter) to 0.045 min^–1^ (with the promoter), as shown in Figure S7. Furthermore, the presence of the turbulence promoter led to lower TOC and COD removals, together with reduced current efficiency, with TOC removals of approximately 33.7% (with TP) and 49.0% (without TP), and COD removals of 80.4% (with TP) and 91.8% (without TP).

Accordingly, the current efficiency decreased from 25.6% for the system without the TP to 22.4% with the TP, indicating that a smaller fraction of the applied current was effectively used for organic oxidation, under the conditions employed. These results shows that promoting mass transport of the reacting species did not necessarily improve the overall degradation performance, indicating that the process was not governed solely by the mass transfer of the organic contaminant, but also by the generation, transport, speciation, and effective utilization of electrochemically produced oxidants.

Although the turbulence promoter was expected to enhance mixing and reduce the thickness of the diffusion layer, its presence also changed the hydrodynamic conditions within the reactor and potentially decreased the effective residence time of electroactive species near the electrode surface. This may contribute to between the pollutant and the electrochemically generated oxidants, limiting the overall oxidation efficiency.

The trends observed with increase of the flow velocity and the use of a turbulence promoter could be attributed to enhanced mass transport under hydrodynamic turbulence conditions, which facilitated interaction between the pollutant and the oxidizing species,^43^ consequently shortening the residence time of electroactive species at the electrode/electrolyte interface. This limited the extent of electrochemical reactions occurring locally in the vicinity of the electrode surface, influencing the overall oxidation efficiency, which appeared to be governed by the balance between oxidant generation and mass transfer conditions.^36,44^ Although similar behavior has been reported for electrochemical systems mediated by active chlorine species, there is still a lack of information available to explain the trade-off between mass transfer and interfacial phenomena, in determining the performance of electrooxidation for pollutant degradation and mineralization. Here, these aspects are discussed further in terms of chlorine speciation and reaction pathways, aiming to understand the role of hydrodynamics in the electrooxidation process. The observed hydrodynamic effects are consistent with the hypothesis that chloride mass transfer, active chlorine generation, and chlorine speciation jointly influence the electrooxidation performance. A conceptual framework describing these interactions is presented in the Supporting Information (Text S2).

### 3.4 Electrochemical Degradation of TMX under Modulated Current

After selecting the best operating conditions (*u* = 0.23 m s^–1^, absence of TP, and pH 9), the electrochemical degradation of TMX was investigated under modulated current (MC) operation, to assess its influence on degradation kinetics and energy efficiency, in comparison with conventional constant current (CC) electrolysis. Overall, the use of modulated current would be expected to significantly decrease the specific energy consumption, by systematically reducing *I*_app_ and, consequently, *U*_cell_, while maintaining high current efficiency.

The modulated-current profile was defined using an exponential decay function, considering the mass-transfer behavior described by eq 4. The applied current was expressed as *I*_app_(*t*) = *I*_0_ exp­(−*k*_mod_ *t*), where *I*_app_ and *I*_0_ are expressed in amperes (A), *t* is expressed in seconds (s), and *k*_mod_ is the current-decay constant (s^–1^). The initial current density was 77.3 mA cm^–2^, determined based on the geometric electrode area of 56 cm^2^, in agreement with the calculated current profile under MC operation (Figure S8). The applied current was progressively decreased according to the exponential profile described by eq 4 until reaching a minimum current density of approximately 5 mA cm^–2^.

The proposed MC strategy was developed based on the chloride mass-transfer-limited current, which governs the generation rate of chlorine-derived oxidants at the anode surface. Accordingly, the applied current profile was designed to track the temporal evolution of chloride transport, thereby minimizing operation under mass-transfer-limited conditions and reducing the fraction of current consumed by parasitic reactions. Under these conditions, a more efficient utilization of the supplied electrical charge is expected, promoting the generation of reactive chlorine species while reducing unnecessary energy consumption.^22,42^ This approach is intrinsically associated with the electrochemical behavior of active MMO anodes. For RuO_2_-based electrodes, pollutant degradation is known to occur predominantly through indirect oxidation mediated by electro-generated active chlorine species, whereas substantially lower oxidation rates are generally observed in sulfate electrolytes due to the absence of these highly reactive mediators.^16,18,45^

Figure 6 shows the evolution of the TMX concentration, TOC, and COD during electrolysis under the MC and CC regimes. Use of the MC mode resulted in faster TMX removal kinetics (*k*_app_ = 0.170 min^–1^), compared to CC operation (*k*_app_ = 0.055 min^–1^) (Figure 6a). This improvement could be attributed to more efficient utilization of the applied current and reduced occurrence of parasitic reactions during the initial stages of the electrolysis. However, for removal of TOC (Figure 6b) and COD (Figure 6c), use of the CC mode provided superior performance, indicating that a sustained high current density favored the oxidation of intermediate species and promoted greater mineralization.

**Figure 6:**
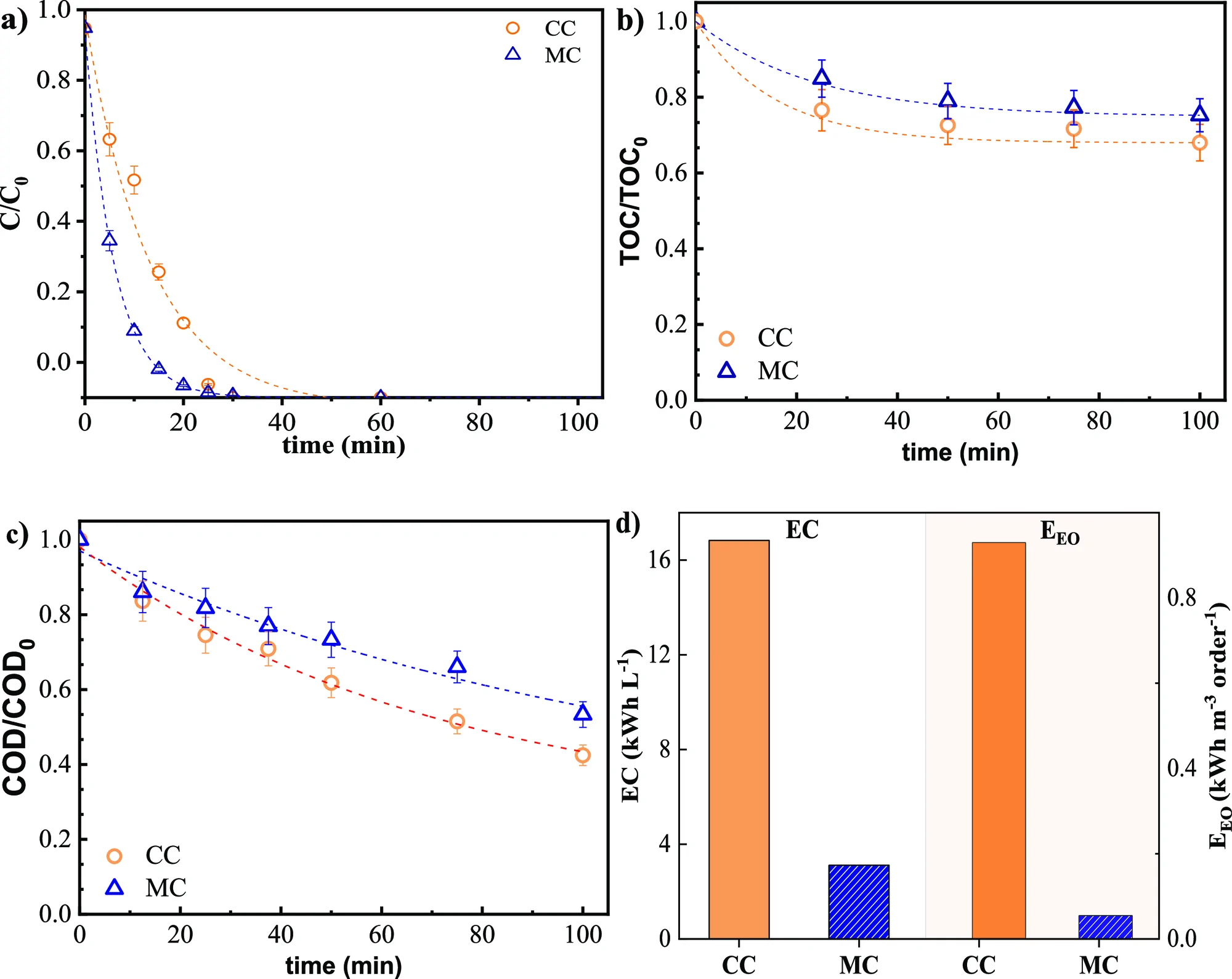
Normalized plots of TMX concentration (a), TOC (b), and COD (c), as a function of electrolysis time, using modulated current (MC) and constant current (CC). (d) EC as a function of electrolysis time. Conditions: Ti/RuO_2_–Sb_2_O_4_–TiO_2_ anode synthesized using CO_2_ laser heating; COD_0_ = 100 mg O_2_ L^–1^; TOC_0_ = 28 mg L^–1^; *V* = 1.4 L; *u* = 0.23 m s^–1^.

After 100 min of electrolysis, TOC and COD removals reached 32.0% and 57.5%, respectively, under CC operation, while MC operation resulted in removals of 24.7% for TOC and 46.7% for COD. Despite the lower mineralization achieved under MC operation, the CE increased from 32.1% to 41.9%, suggesting that adjusting the applied current according to the chloride mass-transfer-limited current improved charge utilization while reducing energy losses. Although the degree of mineralization was lower, energy consumption was significantly reduced under MC operation (Figure 6d), with the specific energy consumption decreasing from 25.5 to 9.81 kWh m^–3^ (∼62% reduction), when the regime was changed from CC to MC. In addition, *E*_EO_ decreased from 1.4 to 0.2 kWh m^–3^ order^–1^, corresponding to an approximately 7-fold reduction (nearly 1 order of magnitude), highlighting MC operation as a highly energy-efficient strategy for the selective degradation of TMX. Although a longer electrolysis time is required, the overall energy demand is significantly lower, resulting in a favorable balance between treatment efficiency and energy consumption.

The behavior observed under MC operation demonstrated that maintaining the applied current close to the limiting current can improve current efficiency and reduce energy consumption in electrochemical oxidation processes. This approach is effective, regardless of whether oxidation proceeds predominantly by means of radical pathways or by mediated oxidation in the bulk solution. Elsewhere Faria et al.^46^ and Verzola et al.,^42^ using a BDD anode for phenol degradation, reported that MC operation significantly decreased the specific energy consumption, although this reduction was accompanied by longer electrolysis times.

In the present work using a Ti/RuO_2_–Sb_2_O_4_–TiO_2_ electrode, the MC strategy provided faster TMX degradation kinetics, whereas CC operation favored higher removals of TOC and COD. These results highlighted that current modulation primarily enhanced the selective oxidation of the contaminant, whereas a sustained high current density was required for greater mineralization. Overall, this study demonstrates the effectiveness of a current modulation approach, based on chloride mass transfer, for electrochemical oxidation using MMO anodes, providing a practical strategy to improve energy efficiency, while maintaining competitive degradation performance.

## 4 Conclusions

Ti/RuO_2_–Sb_2_O_4_–TiO_2_ anodes were synthesized using rapid heating methods and were applied for the electrochemical degradation of TMX in an electrochemical flow reactor, with evaluation of the effects of a turbulence promoter and different current operation modes (continuous and modulated). The rapid heating routes (laser and microwave) produced electrodes with superior physical and electrochemical properties, compared to the electrode obtained using conventional furnace heating. Morphological and electrochemical analyses revealed electroactive surfaces rich in cracks, while accelerated lifetime tests confirmed improved stability of the anode prepared using laser heating. This robust electrode could be successfully employed in a flow reactor configuration.

Reactor performance was significantly influenced by the hydrodynamic conditions. The electrolyte flow velocity had an important effect on TMX degradation, with improved performance observed up to an optimal flow condition (0.23 m s^–1^), highlighting the relevance of hydrodynamic transport in the reactor. Operation without a turbulence promoter resulted in faster degradation kinetics (*k*_app_ = 0.055 min^–1^), compared to the system containing a mesh turbulence promoter (*k*_app_ = 0.045 min^–1^), indicating that the process was mainly limited by oxidant generation, rather than by pollutant mass transfer.

Finally, comparison between the different current operation modes revealed a trade-off between mineralization efficiency and energy demand. Under continuous current operation, TOC and COD removals reached 32.0% and 57.5% after 100 min, while lower removals (24.7% for TOC and 46.7% for COD) were obtained under modulated current (MC) operation, although the latter mode enabled a significant reduction in energy consumption. Although MC promoted faster TMX removal and substantially reduced energy consumption, the comparatively lower TOC removal indicates that rapid parent-compound disappearance was not accompanied by complete mineralization. These results demonstrated that the integration of laser-engineered MMO anodes, optimized hydrodynamics, and dynamic current control can be a promising strategy for the electrochemical treatment of pesticide-contaminated waters. Furthermore, the results support the potential of MC operation as an energy-efficient strategy for the treatment of different classes of organic contaminants. However, its application to other pollutants should be optimized according to their electrochemical reactivity, electrolyte composition in terms of chloride, mass-transfer conditions, and corresponding limiting-current requirements.

## Supplementary Material



## Notes

Additional experimental details and supporting data, including the working hypothesis, determination of the electroactive area and diffusion layer thickness, chlorine speciation diagram, pH evolution, electrochemical characterization, and supplementary degradation results (PDF)

## ∥

Federal University of Ceará, Campus Pici, Avenida Mister Hull, s/n, Antônio Bezerra, 60440900, Fortaleza, CE, Brazil

## References

[1] Wu W., Huang Z.-H., Lim T.-T. (2014). Recent Development of Mixed Metal
Oxide Anodes for Electrochemical Oxidation of Organic Pollutants in
Water. Appl. Catal. A Gen..

[2] Castro, R. S. d. S. ; Dória, A. R. ; Ferreira, M. B. ; Eguiluz, K. I. B. ; Salazar-Banda, G. R. Advancements in Mixed Metal Oxide Anodes for Efficient Electrochemical Treatment of Wastewater. Advances in Chemical Pollution, Environmental Management and Protection; Elsevier, 2024; pp 191–218. 10.1016/bs.apmp.2023.07.001.

[3] Zhou M., Särkkä H., Sillanpää M. (2011). A Comparative Experimental
Study on Methyl Orange Degradation by Electrochemical Oxidation on
BDD and MMO Electrodes. Sep. Purif. Technol..

[4] Duarte J. L. S., Hayat A., Domínguez C. M., Santos A., Cotillas S. (2026). Degradation
Pathways and in Silico Ecotoxicity Assessment of Meropenem By-Products
Generated by Electrochemical Oxidation. J. Environ.
Chem. Eng..

[5] Vasconcelos V. M., Gonzaga I. M. D., Dória A. R., Cordeiro-Junior P. J. M., Lanza M. R. V., Eguiluz K. I. B., Salazar-Banda G. R. (2020). Effects
of Temperature and Heating Method on the Performance of Ti/Ru_0.25_Ir_0.25_Ti_0.50_O_2_ Anodes
Applied toward Bisphenol S Removal. Electrochim.
Acta.

[6] Gonzaga I. M. D., Dória A. R., Castro R. S. S., Souza M. R. R., Rodrigo M. A., Eguiluz K. I. B., Salazar-banda G. R. (2022). Microwave-Prepared
Ti/RuO_2_-IrO_2_ Anodes: Influence of IrO_2_ Content on Atrazine Removal. Electrochim.
Acta.

[7] Dória A. R., Gonzaga I. M. D., Santos G. O. S., Pupo M., Silva D. C., Silva R. S., Rodrigo M. A., Eguiluz K. I. B., Salazar-Banda G. R. (2021). Ultra-Fast
Synthesis of Ti/Ru_0.3_Ti_0.7_O_2_ Anodes
with Superior Electrochemical Properties Using an Ionic Liquid and
Laser Calcination. Chem. Eng. J..

[8] Dória A. R., Gonzaga I. M. D., Santos G. O. S., Almeida C. V. S., Silva D. C., Silva R. S., Romão L. P. C., Sáez C., Salazar-Banda G. R., Eguiluz K. I. B. (2023). Strong Influence
of the Heating Method
on Ti/RuO_2_-TiO_2_ Anode Electrochemical and Photoassisted
Electrochemical Performance. Appl. Catal., B.

[9] Dória A. R., Moratalla A., Almeida C. V. S., Silva R. S., Eguiluz K. I. B., Salazar-Banda G. R., Rodrigo M. A., Saéz C. (2023). Influence
of the Calcination Method and Anode Composition on the Generation
of Disinfectants. Sep. Purif. Technol..

[10] Hayat A., Duarte J. L. S., Cruz-Gómez F., Domínguez C. M., Santos A., Cotillas S. (2025). Electrochemical Degradation
of Levofloxacin
in Synthetic Hospital Effluents: Insights into Operating Parameters,
by-Products Formation and Toxicity. Electrochim.
Acta.

[11] Herraiz-Carboné M., Santos A., Checa-Fernández A., Domínguez C. M., Cotillas S. (2024). Removal of Organochlorine Pollutants from DNAPL-Saturated
Groundwater Using Electrolysis with MMO Anodes. Chem. Eng. J..

[12] Wachter N., Bocchi N., Rocha-Filho R. C. (2021). Use of
a Turbulence Promoter in an
Electrochemical Filter-Press Reactor: Consolidated Evidence of Significant
Enhancement of Organics Mass Transport and Degradation Rates. Sep. Purif. Technol..

[13] Colli A. N., Bisang J. M. (2011). Evaluation of the
Hydrodynamic Behaviour of Turbulence
Promoters in Parallel Plate Electrochemical Reactors by Means of the
Dispersion Model. Electrochim. Acta.

[14] Santos T. É. S., Silva R. S., Carlesi
Jara C., Eguiluz K. I. B., Salazar-Banda G. R. (2014). The Influence
of the Synthesis Method of Ti/RuO_2_ Electrodes on Their
Stability and Catalytic Activity for Electrochemical Oxidation of
the Pesticide Carbaryl. Mater. Chem. Phys..

[15] dos
Reis M. N. G., Ramos Neto A. S., Vasconcelos V. M., Dória A. R., Santos G. O. S., dos Santos E. A., Eguiluz K. I. B., Salazar-Banda G. R. (2021). Ti/Ru_0.7_M_0.3_O_2_ (M = Ir or Ti) Anodes Made by Pechini and Ionic Liquid
Methods: Uneven Catalytic Activity and Stability. J. Electroanal. Chem..

[16] Santos M. O., Santos G. d. O. S., Mattedi S., Griza S., Eguiluz K. I. B., Salazar-Banda G. R. (2018). Influence
of the Calcination Temperature
and Ionic Liquid Used during Synthesis Procedure on the Physical and
Electrochemical Properties of Ti/(RuO_2_)_0.8_–(Sb_2_O_4_)_0.2_ Anodes. J. Electroanal. Chem..

[17] Santos G. O. S., Silva L. R. A., Alves Y. G. S., Silva R. S., Eguiluz K. I. B., Salazar-Banda G. R. (2019). Enhanced
Stability and Electrocatalytic
Properties of Ti/Ru_
*x*
_Ir_1–*x*
_O_2_ Anodes Produced by a New Laser Process. Chem. Eng. J..

[18] Gonzaga I. M. D., Dória A. R., Vasconcelos V. M., Souza F. M., dos Santos M. C., Hammer P., Rodrigo M. A., Eguiluz K. I. B., Salazar-Banda G. R. (2020). Microwave
Synthesis of Ti/(RuO_2_)_0.5_(IrO_2_)_0.5_ Anodes: Improved
Electrochemical Properties and Stability. J.
Electroanal. Chem..

[19] Da
Silva L. M., De Faria L. A., Boodts J. F. C. (2001). Determination
of the Morphology Factor of Oxide Layers. Electrochim.
Acta.

[20] Atkins, P. ; De Paula, J. Physical Chemistry; Macmillan, 2002.

[21] Tang L. (1999). Concentration
Dependence of Diffusion and Migration of Chloride Ions: Part 2. Experimental
Evaluations. Cem. Concr. Res..

[22] Oliveira K. S. G. C., Veroli A. B., Ruotolo L. A. M. (2020). Using
Modulated Current for Energy
Minimization in the Electrochemical Treatment of Effluents Containing
Organic Pollutants. J. Hazard. Mater..

[23] Fernandes C. M., Brillas E., Santos M. C., Garcia-Segura S. (2025). Electro-Fenton
Treatment of Benzophenone-4 Solutions: A Sustainable Approach for
Its Removal Using an Air-Diffusion Cathode. Process Saf. Environ. Prot..

[24] Simões A. J. A., Dória A. R., Vieira D. S., Romão L. P. C., Salazar-Banda G. R., Eguiluz K. I. B. (2023). Electrochemical
Degradation of Ciprofloxacin Using a Coupled 3D Anode to a Microfluidic
Flow-through Reactor. J. Water Process Eng..

[25] Shao D., Yan W., Li X., Yang H., Xu H. (2014). A Highly Stable Ti/TiHx/Sb–SnO2
Anode: Preparation, Characterization and Application. Ind. Eng. Chem. Res..

[26] Shao D., Li X., Xu H., Yan W. (2014). An Improved Stable Ti/Sb–SnO_2_ Electrode with High Performance in Electrochemical Oxidation
Processes. RSC Adv..

[27] Pouladvand I., Asl S. K., Hoseini M. G., Rezvani M. (2019). Characterization and
Electrochemical Behavior of Ti/TiO_2_–RuO_2_–IrO_2_–SnO_2_ Anodes Prepared by
Sol–Gel Process. J. Sol-Gel Sci. Technol..

[28] Guo Y., Zou X., Wei Y., Shu L., Li A., Zhang J., Wang R. (2023). Synthesis of Organic
Hybrid Ruthenium Oxide Nanoparticles for High-Performance
Supercapacitors. Electrochim. Acta.

[29] Santos G. d. O. S., Vasconcelos V. M., da Silva R. S., Rodrigo M. A., Eguiluz K. I. B., Salazar-Banda G. R. (2020). New Laser-Based
Method for the Synthesis
of Stable and Active Ti/SnO_2_–Sb Anodes. Electrochim. Acta.

[30] Audichon T., Morisset S., Napporn T. W., Kokoh K. B., Comminges C., Morais C. (2015). Effect of Adding CeO_2_ to RuO_2_–IrO_2_ Mixed Nanocatalysts:
Activity towards the
Oxygen Evolution Reaction and Stability in Acidic Media. ChemElectroChem.

[31] Jorcin J. B., Orazem M. E., Pébère N., Tribollet B. (2006). CPE Analysis
by Local Electrochemical Impedance Spectroscopy. Electrochim. Acta.

[32] Hoseinieh S. M., Ashrafizadeh F. (2013). Influence
of Electrolyte Composition on Deactivation
Mechanism of a Ti/Ru0.25Ir0.25Ti0.50O2 Electrode. Ionics (Kiel).

[33] Castro R. S. d. S., Dória A. R., Costa F., Mattedi S., Eguiluz K. I. B., Salazar-Banda G. R. (2025). Dipropyl Ammonium Ionic Liquids to
Prepare Ti/RuO2–Sb2O4 Anodes at Different Calcination Temperatures. Environ. Sci. Pollut. Res..

[34] Liu B., Wang C., Chen Y., Ma B. (2019). Electrochemical Behavior
and Corrosion Mechanism of Ti/IrO_2_-RuO_2_ Anodes
in Sulphuric Acid Solution. J. Electroanal.
Chem..

[35] Santos G. d. O. S., Gonzaga I. M. D., Dória A. R., Moratalla A., da Silva R. S., Eguiluz K. I. B., Salazar-Banda G. R., Saez C., Rodrigo M. A. (2020). Testing and Scaling-up of a Novel
Ti/Ru_0.7_Ti_0.3_O_2_ Mesh Anode in a Microfluidic
Flow-through Reactor. Chem. Eng. J..

[36] Panizza M., Cerisola G. (2009). Direct and Mediated Anodic Oxidation
of Organic Pollutants. Chem. Rev..

[37] Dória A. R., Santos G. O. S., Pelegrinelli M. M. S., Silva D. C., de Matos D. B., Cavalcanti E. B., Silva R. S., Salazar-Banda G. R., Eguiluz K. I. B. (2021). Improved 4-Nitrophenol
Removal at Ti/RuO_2_–Sb_2_O_4_–TiO_2_ Laser-Made
Anodes. Environ. Sci. Pollut. Res..

[38] Santos G. O. S., Eguiluz K. I. B., Salazar-Banda G. R., Saez C., Rodrigo M. A. (2020). Photoelectrolysis of Clopyralid Wastes
with a Novel Laser-Prepared MMO-RuO_2_TiO_2_ Anode. Chemosphere.

[39] Wang Z., Li K., Guo J., Yang C., Feng W., Yan L., Zhang Y., Wang J. (2023). Elimination of Pesticide from High
Salinity Wastewater by Electrochlorination Process: Active Chlorine
Species and Scale-up Performance. Sep. Purif.
Technol..

[40] Liu Y., Sun H., Hou J., Bai J., Wang L. A. (2023). Coupling Mechanism
of Anodic Oxygen Evolution Reaction during Organic Pollutants Oxidation. J. Electroanal. Chem..

[41] Zöllig H., Remmele A., Morgenroth E., Udert K. M. (2017). Removal Rates and
Energy Demand of the Electrochemical Oxidation of Ammonia and Organic
Substances in Real Stored Urine. Environ. Sci..

[42] Verzola M. A., Oliveira K. S. G. C., Ruotolo L. A. M. (2022). Optimization of Electrochemical Degradation
of Organics Considering the Simultaneous Effects of Electroactive
Area and Mass Transfer on Current Modulation. J. Environ. Chem. Eng..

[43] Wachter N., Bocchi N., Rocha-Filho R. C. (2021). Use of a Turbulence Promoter in an
Electrochemical Filter-Press Reactor: Consolidated Evidence of Significant
Enhancement of Organics Mass Transport and Degradation Rates. Sep. Purif. Technol..

[44] Cañizares P., García-Gómez J., Lobato J., Rodrigo M. A. (2004). Modeling
of Wastewater Electro-Oxidation Processes Part II. Application to
Active Electrodes. Ind. Eng. Chem. Res..

[45] dos
Anjos Bezerra C. W., de Oliveira Santiago Santos G., Moura de Salles Pupo M., de Andrade Gomes M., Santos da Silva R., Barrios Eguiluz K. I., Salazar-Banda G. R. (2020). Novel Eco-Friendly Method to Prepare
Ti/RuO2–IrO2 Anodes by Using Polyvinyl Alcohol as the Solvent. J. Electroanal. Chem..

[46] Faria L. U. S., Oliveira K. S. G. C., Veroli A. B., Aquino J. M., Ruotolo L. A. M. (2021). Energy Consumption
and Reaction Rate Optimization Combining
Turbulence Promoter and Current Modulation for Electrochemical Mineralization. Chem. Eng. J..

