# Supplementary material for: Laser-Engineered Ti/RuO2–Sb2O4–TiO2 Anodes Enabling Energy-Efficient Electrochemical Degradation of Thiamethoxam in Flow Reactors
Source: ACS Omega. 2026 Sep 28;11(40):61809–22. doi: 10.1021/acsomega.6c07684 (PMC13670305; doi:10.1021/acsomega.6c07684)
Supplement: Supplementary file 1 [file ao6c07684_si_001.pdf]

SUPPLEMENTARY MATERIAL

Laser-engineered $Ti/RuO_2–Sb_2O_4–TiO_2$ anodes enabling energy-efficient electrochemical degradation of thiamethoxam in flow reactors

Aline R. Dória[a,b,c], Géssica O. S. Santos[b,c], Giancarlo R. Salazar-Banda[b,c], Katlin I. B. Eguiluz[b,c], Luís A. M. Ruotolo[a*]

[a] *Federal University of São Carlos, Department of Chemical Engineering, Rod. Washington Luiz, km 235, São Carlos, SP, 13565-905, Brazil*

[b] *Graduate Program in Process Engineering (PEP), Tiradentes University, Aracaju, SE, 49032-490, Brazil*

[c] *Electrochemistry and Nanotechnology Laboratory, Institute of Technology and Research (ITP), Aracaju, SE, 49032-490, Brazil*

* Corresponding author. E-mail address: pluis@ufscar.br (Luís A. M. Ruotolo)

Text S1. Determination of mass transfer parameters and diffusion layer thickness

The diffusion layer thickness (δ) was determined experimentally from mass transport assays using phenol as a probe compound, under diffusion-controlled conditions. The temporal decay of the phenol concentration was described assuming a mass transport-limited regime, in which the concentration decreases exponentially with time, according to Eq. S1, where $C(t)$ is the concentration at time t, C_0 is the initial concentration, k_m is the mass transfer coefficient, A is the electrode area, and V is the electrolyte volume.

$$C(t) = C_0 \exp(-\frac{k_m A}{V} t) \quad \text{(S1)}$$

The experiments were performed using an initial phenol concentration of 100 mg L^{-1} in an aqueous solution containing NaCl (3.6 g L^{-1}) as supporting electrolyte. The total electrolyte volume was 1.4 L and the system was operated under the same hydrodynamic conditions used in the electrooxidation experiments. The electrode area was 0.0056 m^2. The experimental data are shown in Fig. S1.

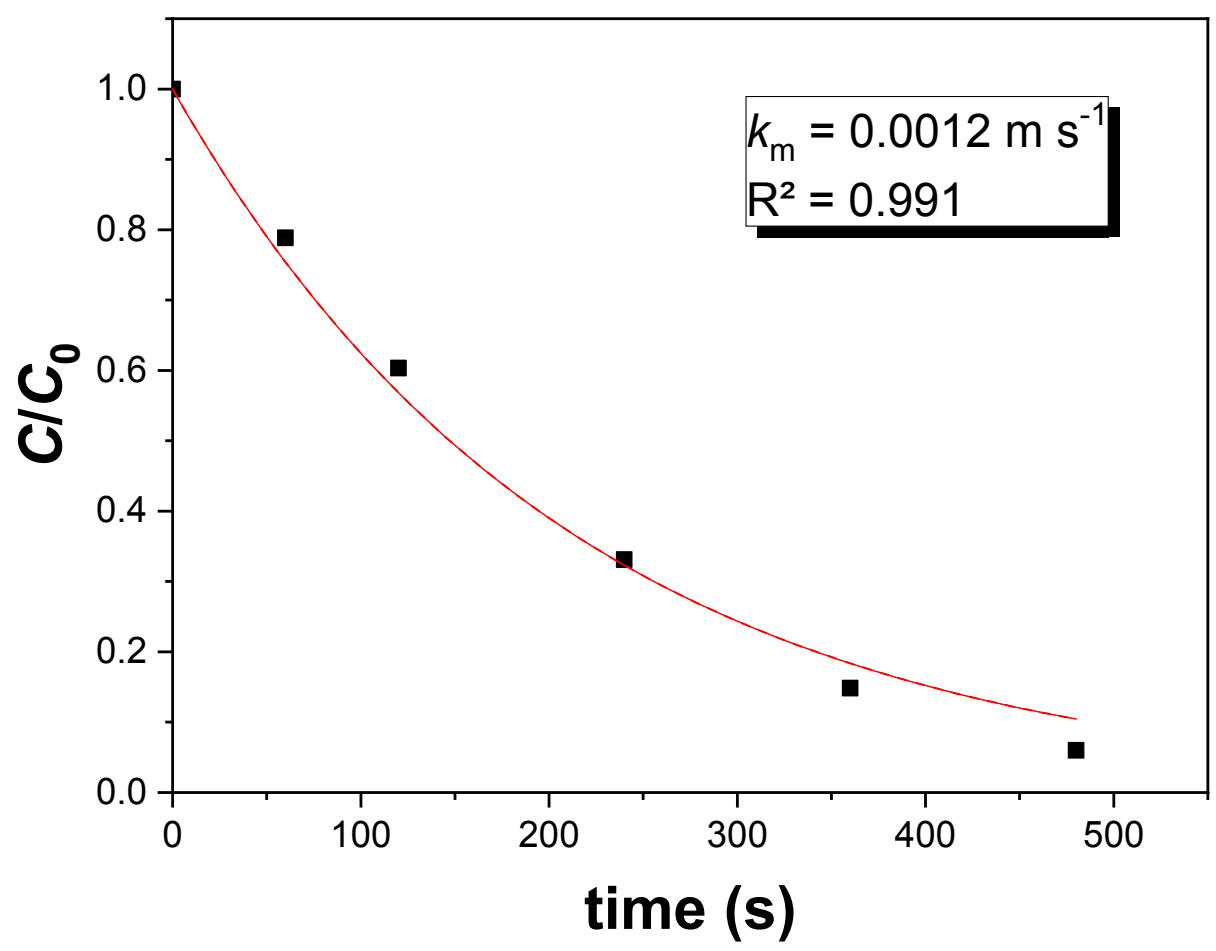

Fig. S1. Experimental decay of the phenol concentration (C/C_0), as a function of time.

The diffusion coefficient of phenol in aqueous solution was taken as 1.28×10^{-9} m^2 s^{-1}. Fitting of the kinetics resulted in a mass transfer coefficient of 1.2×10^{-3} m s^{-1}. The diffusion layer thickness was estimated based on the film diffusion model, using Eq. S2, resulting in a value of $\delta = 1.07 \times 10^{-6}$ m. Here, D_{Cl-} represents the chloride diffusion coefficient (m^2 s^{-1}), whereas $km,_{Cl-}$ represents the chloride mass-transfer coefficient (m s^{-1}).

$$\delta = \frac{D_{phenol}}{k_{m,\,phenol}} \qquad \text{(S2)}$$

The mass transfer coefficient for chloride ions was subsequently estimated (Eq. S3) using the same diffusion layer thickness, assuming identical hydrodynamic conditions, with the diffusion coefficient of chloride in aqueous solution taken as 2.03×10^{-9} m^2 s^{-1}. Under these conditions, the corresponding mass transfer coefficient for chloride ions was calculated as 1.9×10^{-3} m s^{-1}. This approach was justified by the fact that the diffusion layer thickness is primarily governed by the hydrodynamic conditions, rather than the nature of the electroactive species.

$$k_{m,Cl-} = \frac{D_{Cl-}}{\delta} \qquad \text{(S3)}$$

Text S2. Proposed pathways for TMX degradation in Cl^- medium

The following conceptual model represents a proposed hypothesis to interpret the experimental observations obtained under the operating conditions investigated in this study. It is based on the observed experimental trends and established literature on chlorine-mediated electrooxidation. The experimental results indicated that the degradation of TMX in chloride-containing electrolyte was strongly influenced by the hydrodynamic conditions, which affected both mass transfer and the local

reaction environment at the electrode surface. In systems employing active anodes, oxidation is widely reported to predominantly proceed according to mediated pathways involving electrochemically generated chlorine species [2–4], which are formed by the anodic oxidation of chloride ions and are subsequently responsible for the degradation of organic contaminants.

It is well established that anodic reactions, particularly the oxygen evolution reaction, can cause local acidification at the electrode surface (Eq. S4), leading to a pH gradient between the interfacial region and the bulk solution. Therefore, the speciation of electrochemically generated chlorine is expected to differ between these regions. According to chlorine speciation equilibria (Eqs. 10-11), species that are more reactive, such as hypochlorous acid, are favored under acidic conditions, whereas hypochlorite predominates under alkaline conditions. This distinction is particularly relevant, because hypochlorous acid has a significantly higher oxidation potential (1.49 V vs. SHE) than hypochlorite (0.89 V vs. SHE), indicating a higher oxidative capacity. Furthermore, the low current efficiencies observed previously indicate that a significant part of the applied charge may be deviated to the OER, reinforcing that the pH at the electrode surface favors the presence of HOCl.

$$2\,H_2O \rightarrow O_2 + 4\,H^+ + 4e^- \qquad (E^o = 1.23 \text{ V vs. SHE}) \qquad \text{(S4)}$$

Considering the different reaction pathways, shown schematically in Fig. S2, it was possible to propose a conceptual explanation for the TMX electrooxidation behavior as a function of flow velocity, based on the mass-transfer behavior of chloride and TMX under the investigated hydrodynamic conditions. In Fig. S2, $k_{m,Cl}$ and $k_{m,TMX}$ represent the mass transfers of Cl^- and TMX, respectively, from the bulk medium to the anode interface, while $k_{m,HOCl}$ represents the mass transfer of HOCl from the interface to the bulk medium. It should be highlighted that the mass transfer coefficients (Eq. S2) are directly influenced by the thickness of the diffusion layer (δ), which, in turn, depends on the hydrodynamic conditions. Hence, increasing the flow velocity or using a TP would promote the compression of δ and, consequently, the enhancement of k_m.

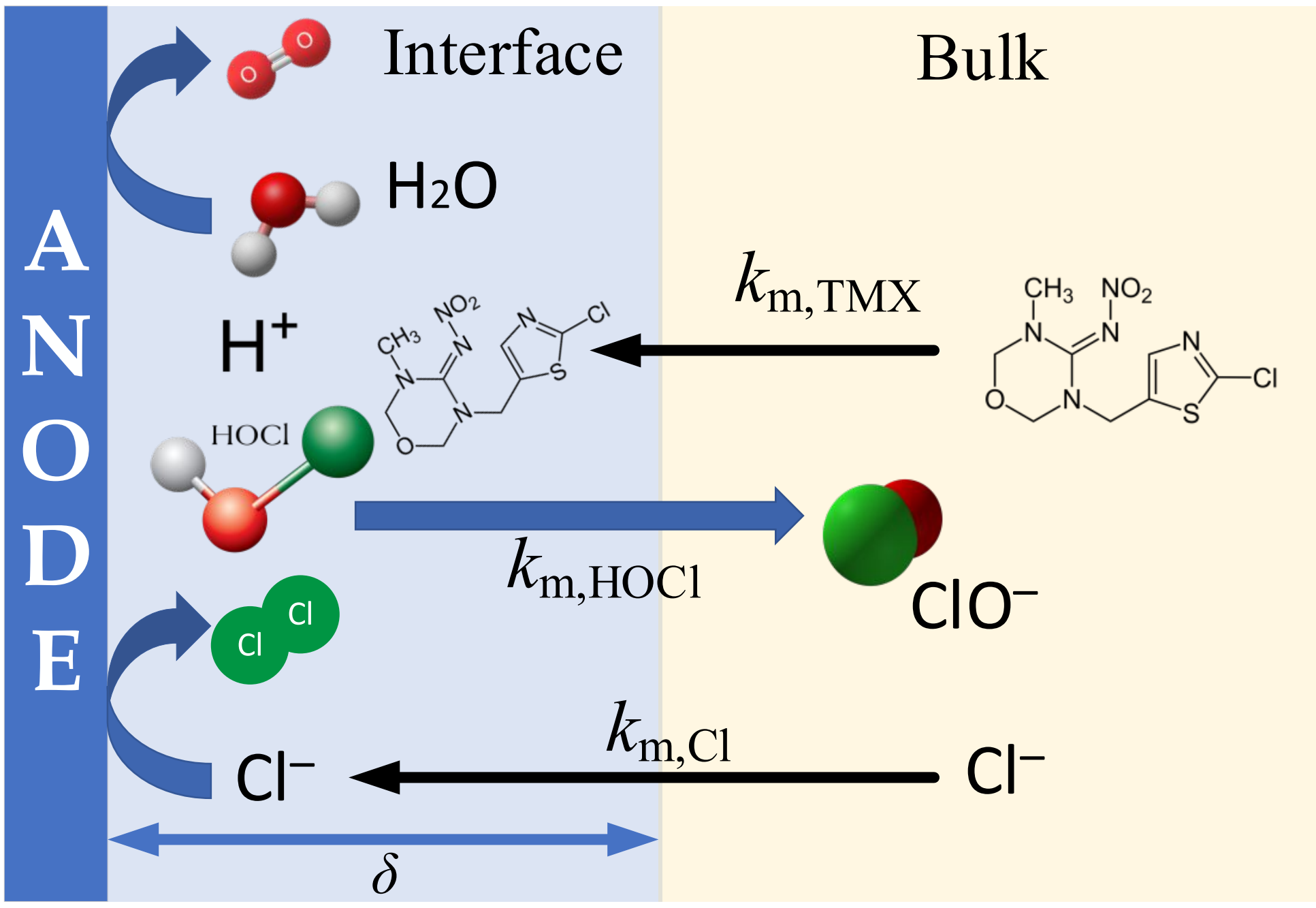

Fig. S2. Proposed oxidation pathways for TMX degradation in a chloride medium. The scheme highlights the role of mass transfer and the influence of local pH on chlorine speciation.

In the proposed reaction pathways, described by Eqs. S5-S7, two distinct regions can be recognized: 1) the anode/electrolyte interface, corresponding to the volume of the diffusion layer ($V_d = \delta A$); and 2) the bulk region, corresponding to V_b. In the volume close to the interface, predominance of the HClO active chorine species is expected, due to the acidification of the interface (Eq. 4), so its concentration ($C_{HClO,i}$), according to Eq. S5, depends on the mass transfer of Cl^- to the interface, where it is oxidized to chorine and converted to HOCl, as shown in the speciation diagram (Fig. S3).

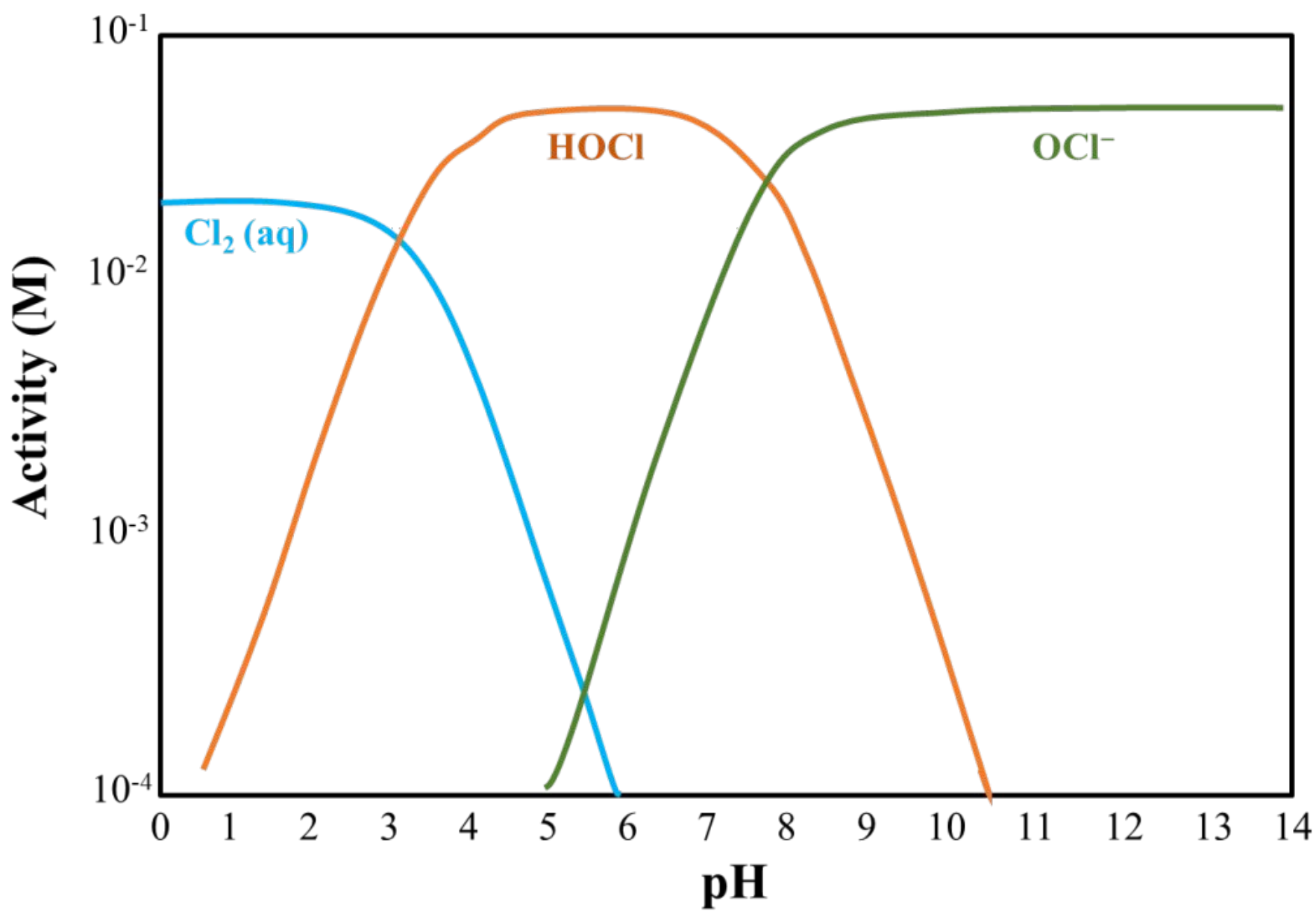

Fig. S3. Distribution of chlorine oxidizing species, as a function of pH, at 25 °C (adapted from [1]).

At the interface, HOCl can react with TMX, according to the homogeneous kinetic expression $r_1 = k_1 C_{\mathrm{HClO,V_d}} C_{\mathrm{TMX,V_d}}$, or be transported to the bulk medium, according to the mass transfer kinetics ($k_{\mathrm{m,HOCl}}\ A C_{\mathrm{HClO,i}}$). The kinetic constant, k_1, depends on the concentrations of HClO and TMX in the volume V_d. On the other hand, the concentration of the less oxidizing ClO^- species in the bulk volume (V_b), expressed in Eq. S6, is affected by the concentration of the HClO, arriving by mass transfer from the interface to the bulk volume, and the homogeneous reaction ($r_2 = -\ k_2 C_{\mathrm{ClO,V_b}}$ $C_{\mathrm{TMX,V_b}}$). Finally, Eq. S7 expresses the TMX concentration in the electrolyte volume (V), showing that the TMX concentration depends on complex oxidation pathways involving mass transfer, homogeneous reactions, and chlorine speciation.

$$V_{\mathrm{d}} \frac{dC_{\mathrm{HClO,i}}}{dt} = -\ k_{\mathrm{m,Cl}} A \left(C_{\mathrm{Cl,b}} - C_{\mathrm{Cl,i}} \right) + k_{\mathrm{m,HOCl}} A C_{\mathrm{HClO,i}} - k_1 C_{\mathrm{HClO,V_d}} C_{\mathrm{TMX,V_d}} \quad \text{(S5)}$$

$$V_{\mathrm{b}} \frac{dC_{ClO}}{dt} = k_{\mathrm{m,HClO}} A C_{\mathrm{HClO,i}} - k_2 C_{\mathrm{ClO,V_b}} C_{\mathrm{TMX,V_b}} \quad \text{(S6)}$$

$$V \frac{dC_{\mathrm{TMX}}}{dt} = -k_{\mathrm{m,TMX}} A \left(C_{\mathrm{TMX,b}} - C_{\mathrm{TMX,i}} \right) - k_1 C_{\mathrm{HClO,V_d}} C_{\mathrm{TMX,V_d}} - k_2 C_{\mathrm{ClO,V_b}} C_{\mathrm{TMX,V_b}} \quad \text{(S7)}$$

The proposed model was then used to explain the effects of flow velocity and turbulence promoter on the TMX degradation observed in the electrooxidation experiments. As the flow velocity increased from 0.13 to 0.23 m s^{-1}, the mass transfer of chloride and TMX increased, enhancing their transport towards the anode surface. Under these conditions, the combination of improved mass transfer and the locally acidified interfacial environment would be expected to favor the generation of the more reactive HOCl chlorine species, resulting in enhanced degradation kinetics and mineralization efficacy.

On the other hand, a further increase in flow velocity led to a different behavior. In this case, parallel to the mass transfer improvement, the residence time of electroactive species within the diffusion layer was shortened. With increase of the flow velocity or use of the TP, the increased hydrodynamic turbulence compressed δ, so $k_{\mathrm{m,Cl}}$ was favored and more HClO was produced. However, the thinner diffusion layer also improved the transfer of HClO from the interface to the bulk volume, where it was converted to the less oxidizing ClO^- species. Consequently, a complex trade-off between mass transfer and homogeneous reactions (represented by k_1 and k_2) would be established, which would determine the overall TMX degradation kinetics.

In the case of increasing the flow velocity from 0.23 to 0.30 or 0.37 m s^{-1} (or using the TP), the shorter residence time of HClO at the interface, provided by an increase of $k_{\mathrm{m,HClO}}$ and lower V_d (due to δ compression), appeared to limit the contact between TMX and this more oxidizing chlorine species formed under the locally acidic conditions found at the electrode/electrolyte interface. In parallel, a larger fraction of HClO was transported to the bulk volume, where the pH remained alkaline and chlorine speciation shifted towards the less reactive species (OCl^-), which had lower

oxidation potential and reduced reactivity towards TMX oxidation. This shift resulted in a decrease of the TMX degradation rate, as shown in Fig. 5(a) in the manuscript.

In summary, the overall electrochemical degradation of TMX could be described as a process governed by a balance among (i) mass transfer of chloride ions and organic molecules to the electrode surface, (ii) generation of oxidizing species under a locally acidified interface condition, and (iii) effective contact between oxidant and pollutant. The existence of an optimal flow velocity reflected the competition among these different mass transfer phenomena occurring at the electrode interface. Although the formation of higher oxidation state chlorine species (e.g., chlorate and perchlorate) has been reported to be limited for active MMO anodes under similar operating conditions, these species were not quantified in the present study. Therefore, they were not included in the proposed conceptual interpretation [4–6]. Therefore, these species were not considered in the analysis. Finally, it should be highlighted that it is a hypothesized mechanism, suitable to explain the oxidation trends observed for TMX oxidation, but that requires further insights for validation based on experimental results, which will be addressed in future works.

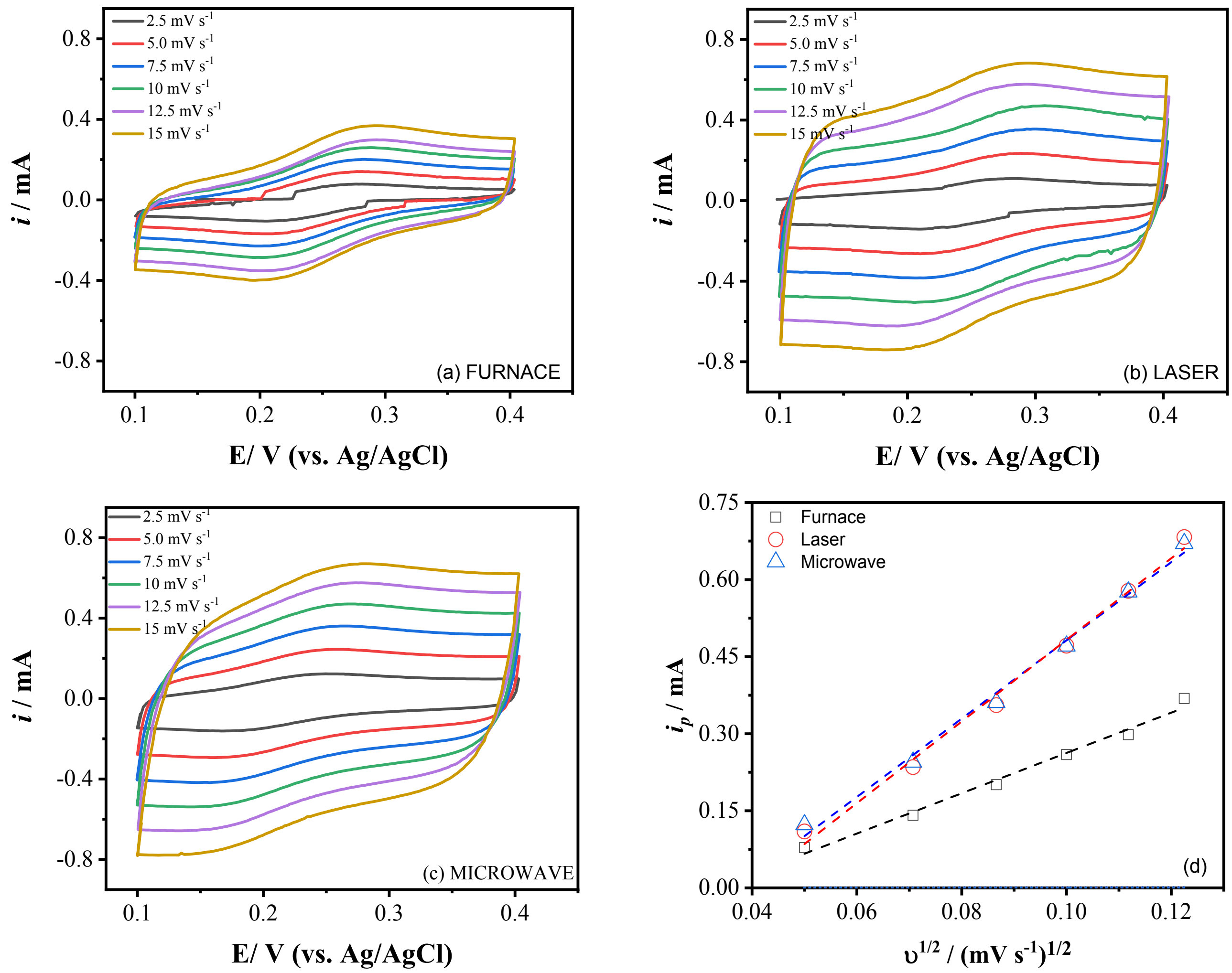

Fig. S4. Cyclic voltammograms recorded in the potential window 0.1-0.4 V, at different scan rates (2.5-15 mV s^{-1}), for the anodes prepared using the furnace **(a)**, laser **(b)**, and microwave **(c)** calcination

procedures. **(d)** Anodic peak current as a function of $v^{1/2}$ for all the anodes. Electrolyte: 1.0 mM $K_3[Fe(CN)_6]$ and 3.7 g L^{-1} KCl.

Table S1. Parameters obtained from linear fitting of the electrochemical data for the Ti/RuO_2-Sb_2O_4-TiO_2 anodes synthesized using the different heat treatments, including intercept, slope, square determination coefficient (R^2), and electroactive area (A_E). Electrolyte: 1.0 mM $K_3[Fe(CN)_6]$ and 3.7 g L^{-1} KCl.

Parameter	Furnace	Laser	Microwave
Intercept	-1.3×10^{-4}	-3.1×10^{-4}	-2.8×10^{-4}
Slope	0.00392	0.00795	0.00762
R^2	0.98	0.99	0.99
A_E (cm^2)	5.29	10.7	10.3

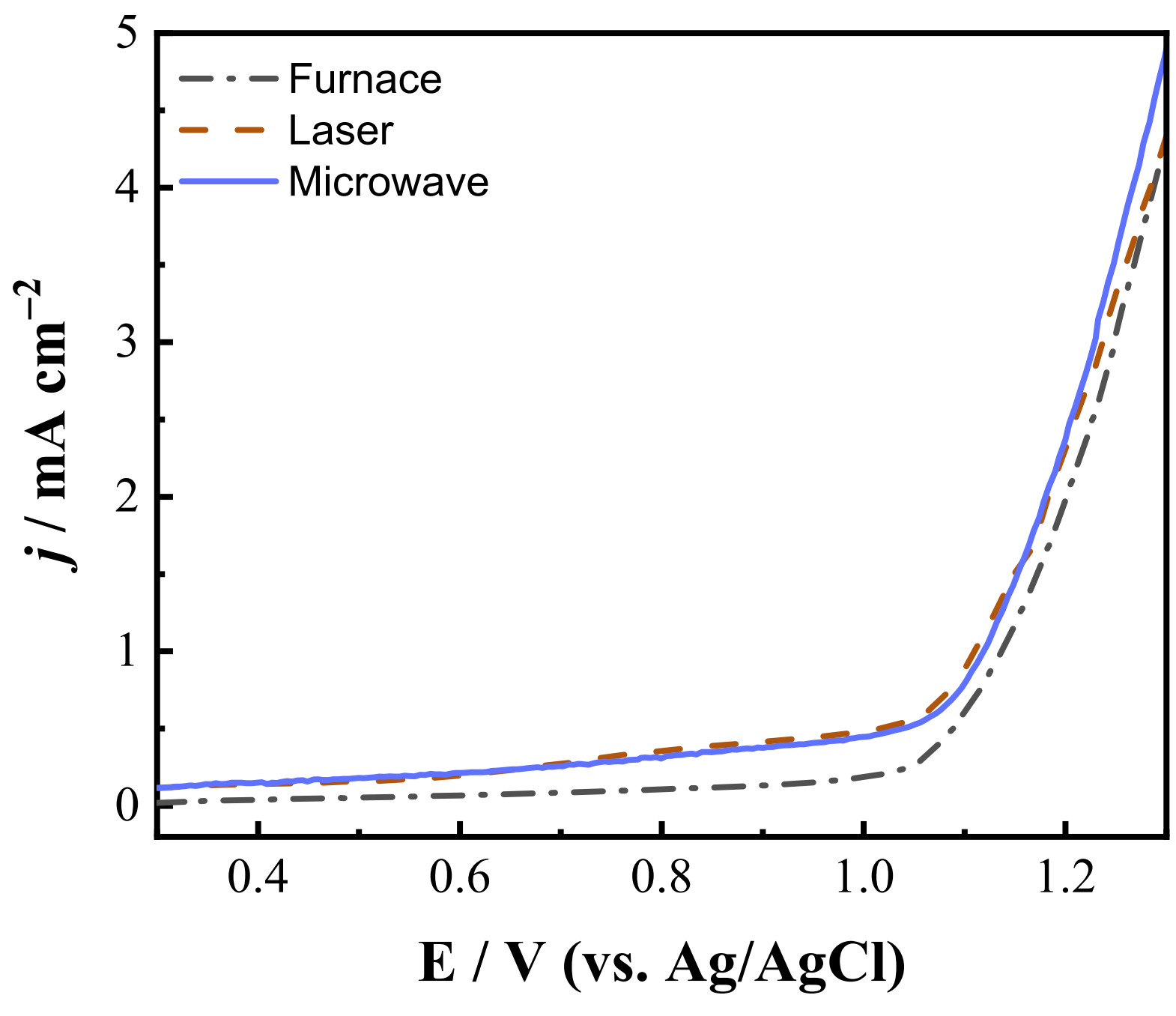

Fig. S5. Linear sweep voltammetry profiles recorded at 10 mV s^{-1} for the Ti/RuO_2-Sb_2O_4-TiO_2 anodes synthesized using the furnace, laser, and microwave heat treatments.

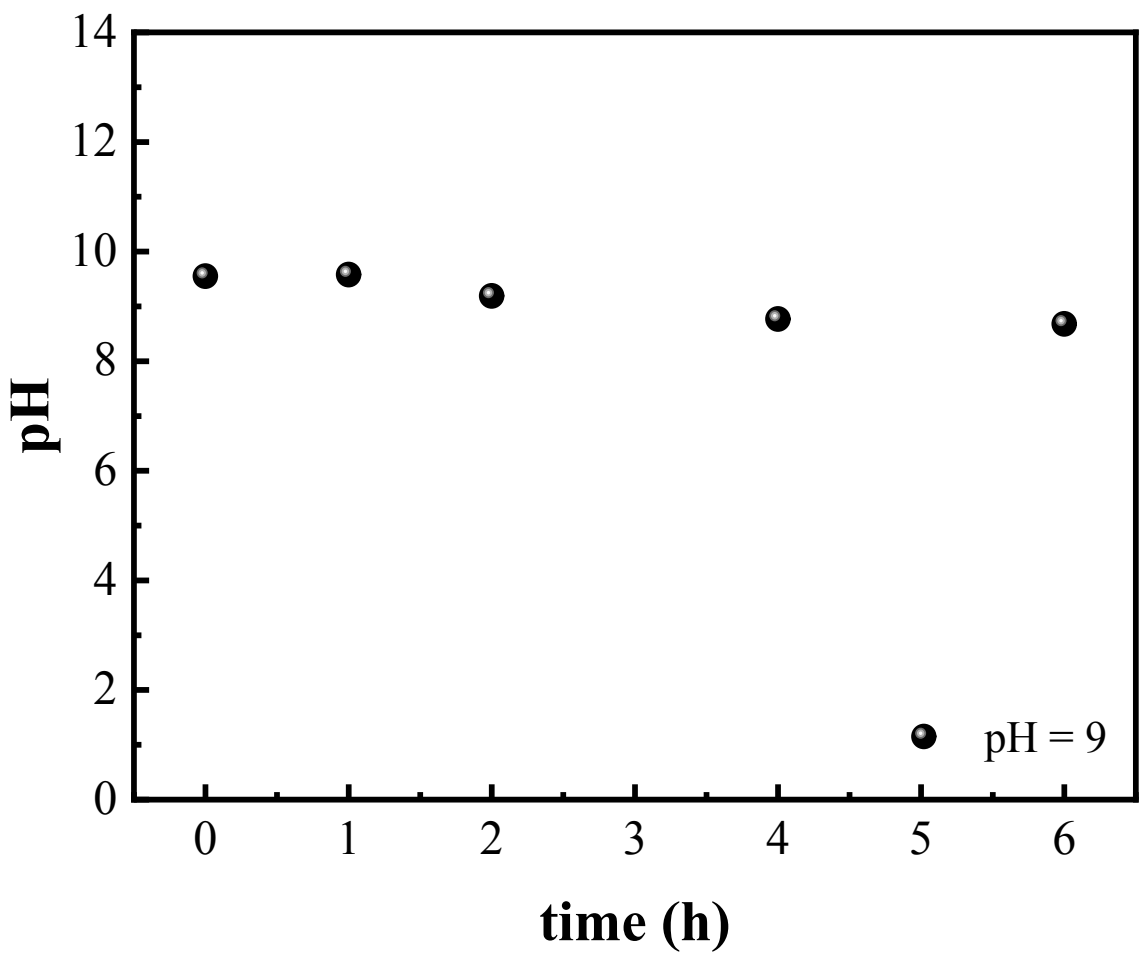

Fig. S6. Variation of pH as a function of electrolysis time, under uncontrolled conditions (pH_0 = ~9). Conditions: $Ti/RuO_2–Sb_2O_4–TiO_2$ anode synthesized using CO_2 laser heating; COD_0 = ~100 mg O_2 L^{-1}; TOC_0 = 28 mg L^{-1}; j = 50 mA cm^{-2}; V = 1.4 L; NaCl = 3.7 g L^{-1}; u = 0.23 m s^{-1}.

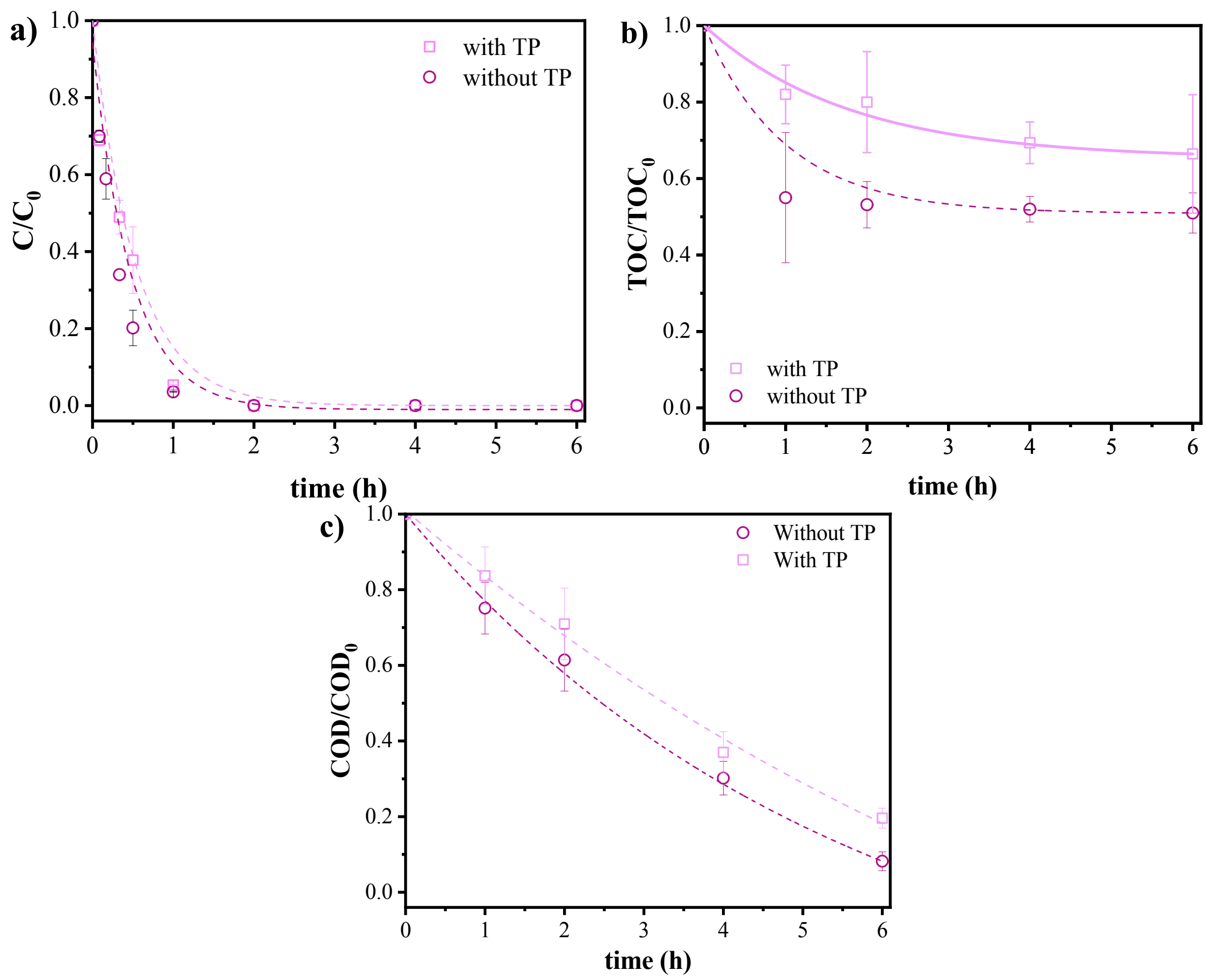

Fig. S7. Normalized plots for TMX removal **(a)**, TOC **(b)**, and COD **(c)**, as a function of time, for different flow velocities, for electrooxidation performed in the presence of a turbulence promoter. The lines represent the fitted pseudo-first order model. Conditions: $Ti/RuO_2–Sb_2O_4–TiO_2$ anode synthesized using CO_2 laser heating; COD_0 = ~100 mg O_2 L^{-1}; TOC_0 = 28 mg L^{-1}; pH_0 = ~9; j = 50 mA cm^{-2}; V = 1.4 L; NaCl = 3.7 g L^{-1}; u = 0.23 m s^{-1}.

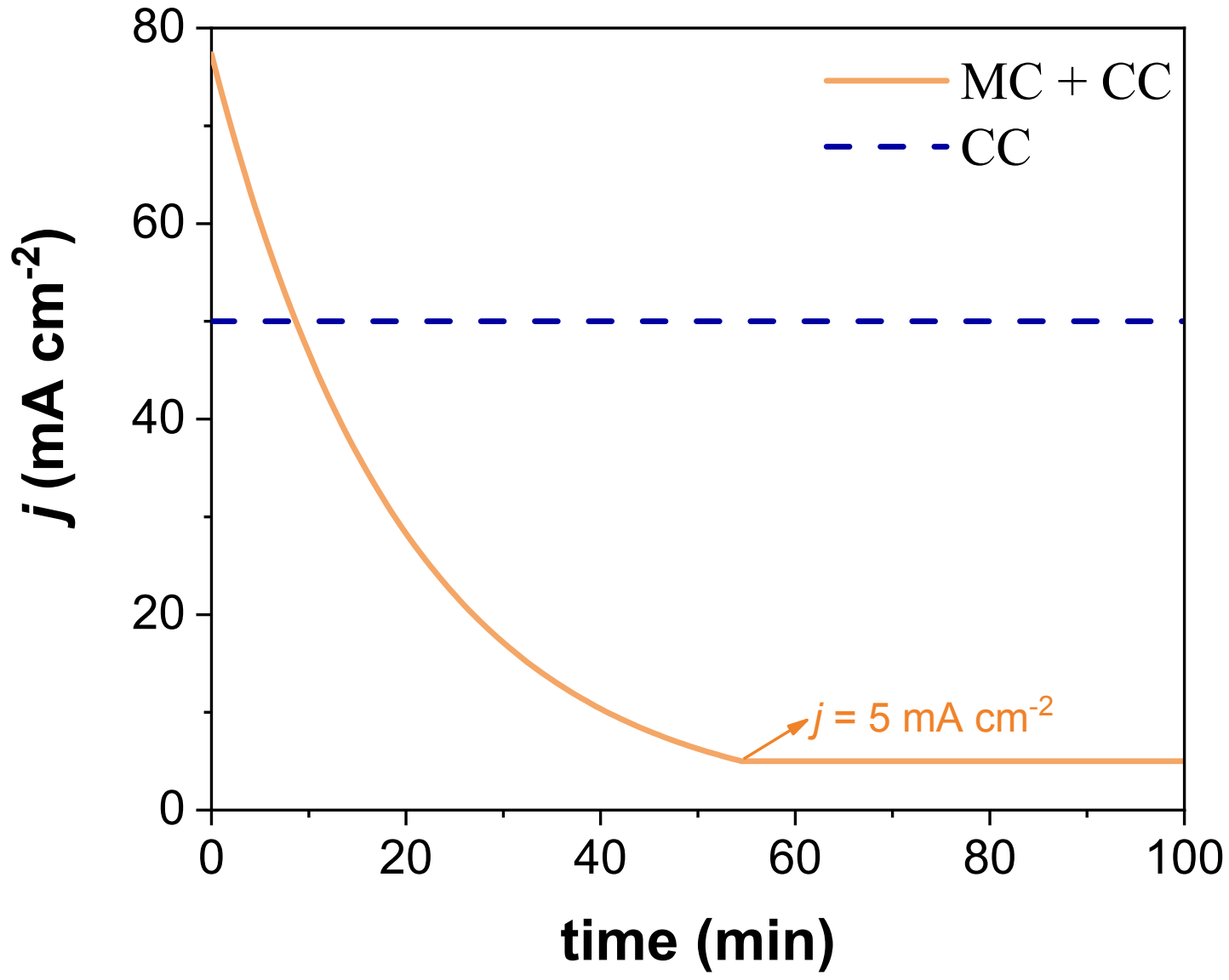

Fig. S8. Time-dependent current density profiles applied during electrochemical oxidation under modulated current (MC + CC) and constant current (CC) operation.

Table S2. Kinetic parameters obtained from pseudo-first order fitting for TMX, TOC, and COD removal under different operational conditions: flow velocity, presence or absence of turbulence promoter, pH, constant current (CC), and modulated current (MC).

	**Flow velocity (m s^{-1})**				**Turbulence promoter**		**pH**		**Current mode**	
	0.13	0.23	0.30	0.37	With	Without	pH 9	pH 4	CC	MC
**TMX**										
k_{app}	0.0186	0.055	0.021	0.022	0.045	0.055	0.055	0.12	0.055	0.1713
R^2	0.926	0.999	0.910	0.996	0.975	0.999	0.999	0.991	0.999	0.999
**TOC**										
k_{app}	0.0009	0.0013	0.0013	0.0013	0.0009	0.0013	0.0013	0.0034	0.0045	0.0032
R^2	0.978	0.935	0.916	0.980	0.982	0.935	0.935	0.922	0.913	0.9598
**COD**										
k_{app}		0.0062	0.0042		0.0043	0.0062	0.0062	0.0043	0.009	0.0062
R^2		0.971	0.998		0.988	0.971	0.971	0.988	0.993	0.986

REFERENCE

(1) Carneiro, M. A.; de Kroon, E.; Vital, B.; Pereira, S. P.; Agostinho, L. L. F. Electrochemical Process of Chlorination and Energy Generation as Viable Alternatives for SWRO Brine Valorization. *Desalination* **2024**, *586*, 117875. https://doi.org/10.1016/J.DESAL.2024.117875.

(2) De Moura, D. C.; De Araújo, C. K. C.; Zanta, C. L. P. S.; Salazar, R.; Martínez-Huitle, C. A. Active Chlorine Species Electrogenerated on $Ti/Ru_{0.3}Ti_{0.7}O_2$ Surface: Electrochemical Behavior, Concentration Determination and Their Application. *Journal of Electroanalytical Chemistry* **2014**, *731*, 145–152. https://doi.org/10.1016/j.jelechem.2014.08.008.

(3) Wang, Z.; Li, K.; Guo, J.; Yang, C.; Feng, W.; Yan, L.; Zhang, Y.; Wang, J. Elimination of Pesticide from High Salinity Wastewater by Electrochlorination Process: Active Chlorine Species and Scale-up Performance. *Sep. Purif. Technol.* **2023**, *306*, 122572. https://doi.org/10.1016/J.SEPPUR.2022.122572.

(4) Dória, A. R.; Gonzaga, I. M. D.; Santos, G. O. S.; Almeida, C. V. S.; Silva, D. C.; Silva, R. S.; Romão, L. P. C.; Sáez, C.; Salazar-Banda, G. R.; Eguiluz, K. I. B. Strong Influence of the Heating Method on Ti/RuO_2-TiO_2 Anode Electrochemical and Photoassisted Electrochemical Performance. *Appl. Catal. B* **2023**, *339*. https://doi.org/10.1016/j.apcatb.2023.123092.

(5) Antonelli, R.; Malpass, G. R. P.; Carlos da Silva, M. G.; Vieira, M. G. A. Ofloxacin Degradation in Chloride-Containing Medium by Photo-Assisted Sonoelectrochemical Process Using a Mixed Metal Oxide Anode. *J. Environ. Chem. Eng.* **2022**, *10* (2), 107174. https://doi.org/10.1016/j.jece.2022.107174.

(6) Montes, I. J. S.; Silva, B. F.; Aquino, J. M. On the Performance of a Hybrid Process to Mineralize the Herbicide Tebuthiuron Using a DSA® Anode and UVC Light: A Mechanistic Study. *Appl. Catal. B* **2017**, *200*, 237–245. https://doi.org/10.1016/j.apcatb.2016.07.003.
